# Lignin‐Based Photothermal Materials: Bridging Sustainability and High‐Efficiency Energy Conversion

**DOI:** 10.1002/advs.202501259

**Published:** 2025-04-25

**Authors:** Zhiwen Sun, Changyou Shao, Sanwei Hao, Jifei Zhang, Wenfeng Ren, Bing Wang, Lingping Xiao, Hanhui Lei, Terence X. Liu, Zhanhui Yuan, Run‐cang Sun

**Affiliations:** ^1^ Liaoning Key Laboratory of Lignocellulose Chemistry and BioMaterials Liaoning Collaborative Innovation Center for Lignocellulosic Biorefinery College of Light Industry and Chemical Engineering Dalian Polytechnic University Dalian 116034 China; ^2^ College of Materials Engineering Fujian Agriculture and Forestry University Fuzhou 350002 China; ^3^ School of Materials Science and Engineering Shandong University of Technology Zibo 255000 China; ^4^ Department of Mechanical and Construction Engineering Northumbria University Newcastle upon Tyne NE1 8ST UK

**Keywords:** environmental sustainability, lignin, photothermal conversion, photothermal effect, renewable energy

## Abstract

Photothermal materials can effectively absorb light and convert it into heat, providing sustainable solutions to mitigate environmental pollution and energy shortages. Compared to traditional photothermal materials, lignin has garnered significant attention due to its wide availability, low cost, biocompatibility, renewability, and sustainability. Consequently, lignin‐based materials are considered ideal candidates for the development of eco‐friendly photothermal systems, aligning well with the increasing demand for sustainable energy solutions. This review discusses the potential of lignin‐based photothermal materials, highlighting their unique molecular structure and the photothermal properties imparted by their aromatic rings, which facilitate effective energy conversion through non‐radiative vibrational relaxation. Discussed the latest advances in the applications of lignin photothermal materials in photothermal drive, solar desalination, and biomedicine. Despite the significant potential of lignin, challenges such as structural variability, long‐term stability, and scalability remain critical. This paper integrates recent progress and proposes strategies to optimize the photothermal performance of lignin‐based materials, while emphasizing important directions for sustainable development, thereby providing a roadmap to fully realize the potential of lignin in next‐generation green technologies.

## Graphical abstract

This review provides new perspectives on lignin‐based photothermal materials, emphasizing their molecular structure for efficient energy conversion. It summarizes mechanisms and strategies for improving photothermal efficiency in applications of photothermal actuation, desalination, and biomedical therapies. Challenges in stability, scalability, and structural variability are highlighted, with future prospects proposed for optimizing performance and advancing sustainable technologies.

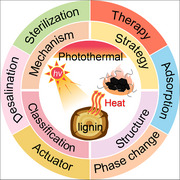

## 1 Introduction

Environmental pollution and energy scarcity are urgent challenges driven by rapid population growth and industrialization, which have led to the overexploitation of fossil fuels, resource depletion, and ecosystem degradation.^[^ ^1^, ^2^ ^]^ The production of petroleum‐based chemicals further exacerbates greenhouse gas emissions and environmental pollution.^[^ ^3^ ^]^ The “dual‐carbon policy” further emphasizes the urgency for change in the energy and environmental sectors.^[^ ^4^ ^]^ Tackling these issues requires the development of clean, renewable energy sources and sustainable materials.

Among renewable energy forms, solar energy is favored for its abundance and cleanliness.^[^ ^5^ ^]^ Photothermal materials, a class of functional materials capable of efficiently absorbing sunlight and converting it into thermal energy, hold significant promise for advancing solar energy utilization.^[^ ^6^ ^]^ Different photothermal conversion mechanisms allow for the classification of photothermal materials into three primary types: plasmonic localized heating, non‐radiative relaxation in semiconductors, and thermal vibrations within molecules.^[^ ^7^ ^]^ However, traditional photothermal materials such as metallic nanoparticles and semiconductor materials suffer from high costs, complex fabrication processes, and poor environmental compatibility, significantly hindering their practical applications. To overcome these challenges, identifying cost‐effective alternative materials becomes crucial. Lignin, due to its low cost and potential for photothermal conversion, stands out as an ideal candidate.

Lignin, second only to cellulose as a renewable biomass resource, represents one of the most abundant natural polymers globally.^[^ ^8^ ^]^ Annual lignin production from the pulping industry alone reaches ≈50 million tons, underscoring its significant availability and potential for sustainable utilization.^[^ ^9^ ^]^ Despite the abundant availability of lignin resources, a substantial portion undergoes incineration or disposal, leading to significant environmental pollution and considerable energy waste.^[^ ^10^, ^11^ ^]^ The development of high‐value applications for lignin thus represents an urgent challenge in contemporary research. Compared to traditional photothermal materials such as gold nanoparticles and titanium oxide, lignin offers a more economical, efficient, and eco‐friendly alternative, which enhances its appeal in scalable applications. Therefore, lignin as a photothermal material is receiving increasing attention. Chen's research group^[^ ^12^ ^]^ investigated the photothermal effect of alkali lignin nanoparticles (L‐NPs) and found that L‐NPs exhibit strong absorption in the solar spectrum, with a stable photothermal conversion efficiency of 22% under simulated solar radiation of 100 mW cm^−2^. To further improve the efficiency, Shao et al.^[^ ^13^ ^]^ explored the demethylation modification of lignin. The modified lignin exhibited a reduced molecular weight and increased phenolic hydroxyl content, resulting in more compact *π–π* stacking, which increased the photothermal efficiency of the modified lignin to 43.2%. These results indicate that lignin holds great potential as a photothermal material.

**Figure 1:**
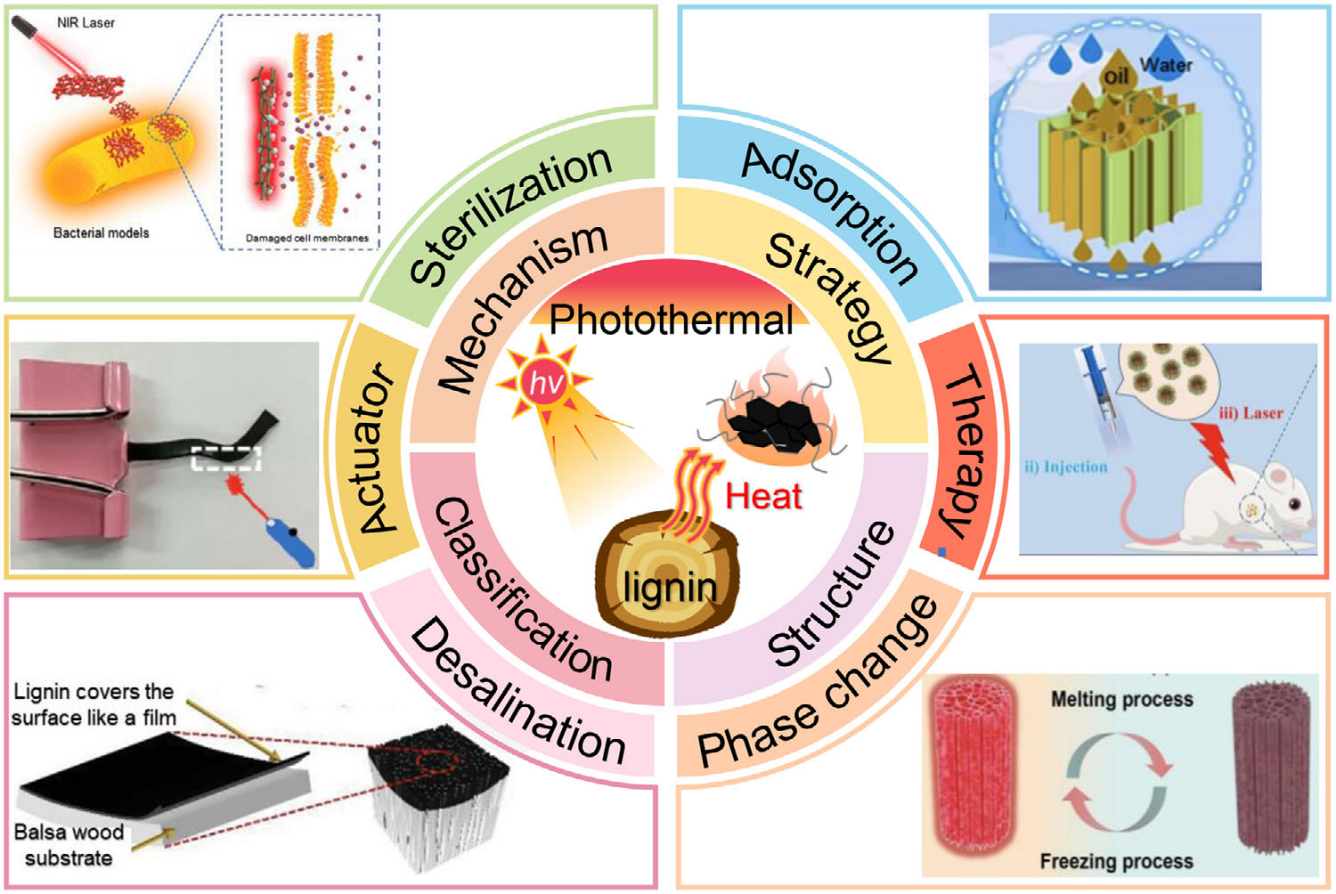
Overview of major applications of lignin‐based photothermal materials: actuation,^[^ ^25^ ^]^ Copyright 2022, American Chemical Society seawater desalination,^[^ ^16^ ^]^ Copyright 2023, Wiley. photothermal therapy,^[^ ^26^ ^]^ Copyright 2024, Elsevier sterilization,^[^ ^19^ ^]^ Copyright 2022, American Chemical Society. adsorption^[^ ^20^ ^]^ Copyright 2024, Elsevier and phase change.^[^ ^27^ ^]^ Copyright 2024, Elsevier.

**Figure 2:**
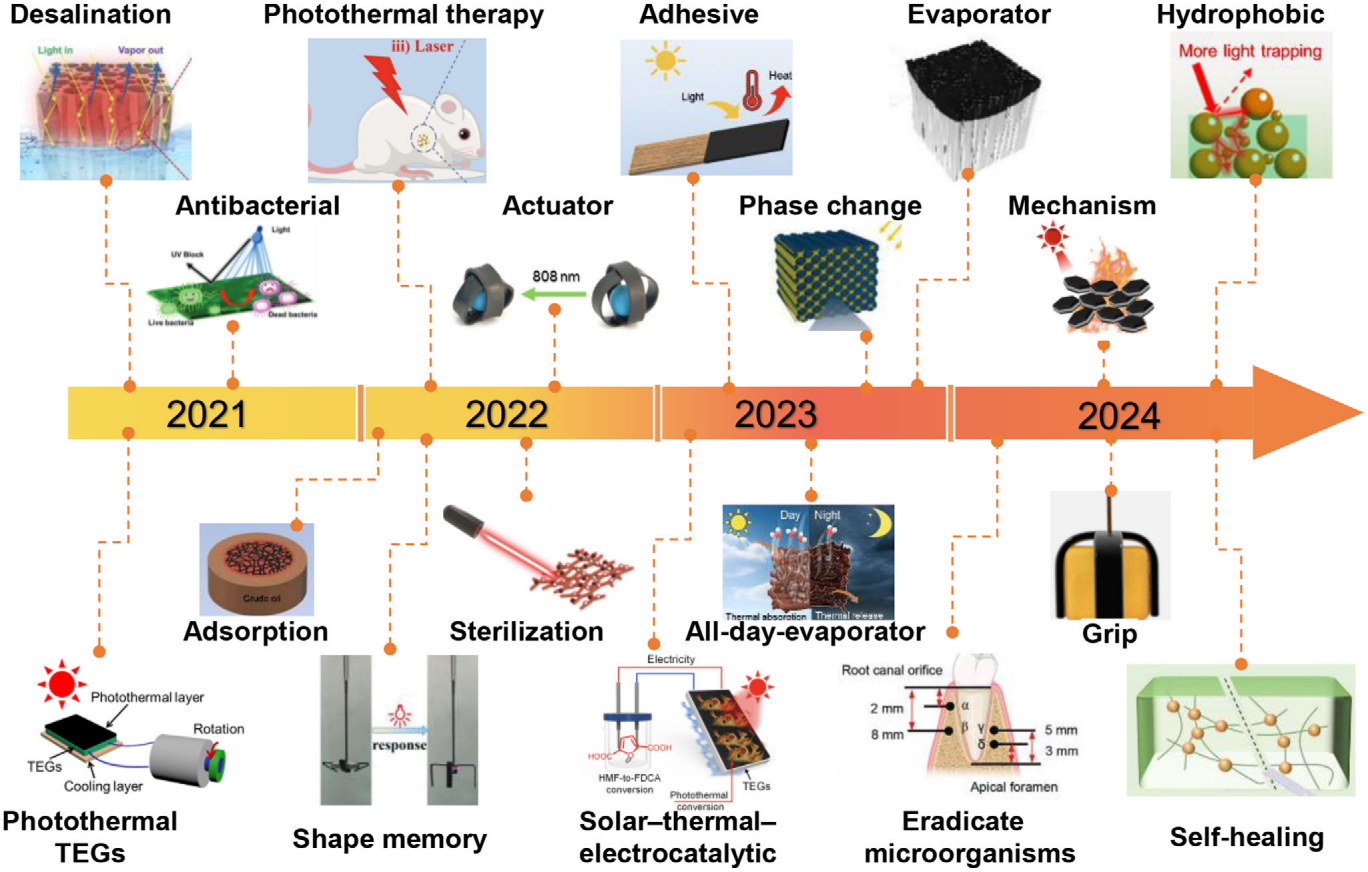
A concise timeline of photothermal materials for lignin and its derivatives. Desalination,^[^ ^28^ ^]^ Copyright 2022, Elsevier antibacterial properties,^[^ ^29^ ^]^ Copyright 2021, Royal Society of Chemistry. photothermal thermoelectric generation,^[^ ^12^ ^]^ Copyright 2022, Elsevier waste oil adsorption,^[^ ^30^ ^]^ Copyright 2022, Elsevier photothermal therapy,^[^ ^26^ ^]^ Copyright 2023, Elsevier shape memory,^[^ ^14^ ^]^ Copyright 2022, Royal Society of Chemistry. photothermal sterilization,^[^ ^31^ ^]^ Copyright 2022, American Chemical Society. actuator,^[^ ^25^ ^]^ Copyright 2022, American Chemical Society. adhesives,^[^ ^32^ ^]^ Copyright 2023, Royal Society of Chemistry. solar‐thermal‐electrocatalytic,^[^ ^5^ ^]^ Creative Commons Attribution 3.0 Unported Licence. All‐day evaporators,^[^ ^33^ ^]^ Copyright 2023, Elsevier hydrophobicity,^[^ ^34^ ^]^ Copyright 2024, Elsevier microorganism eradication,^[^ ^18^ ^]^ Copyright 2023, Wiley. mechanisms,^[^ ^35^ ^]^ Copyright 2024, Royal Society of Chemistry. gripping,^[^ ^15^ ^]^ Copyright 2024, Elsevier wooden evaporators,^[^ ^16^ ^]^ Copyright 2023, Wiley. self‐healing.^[^ ^22^ ^]^ Copyright 2024, Wiley.

Lignin‐based photothermal materials have achieved significant advancements across diverse fields (**Figure** **1**). In photothermal actuation, researchers have harnessed the exceptional photothermal conversion performance of lignin to fabricate light‐driven actuators and successfully achieve remote control.^[^ ^14^, ^15^ ^]^ Lignin‐based photothermal materials have been used to prepare high‐performance seawater desalination devices and effectively enhance desalination efficiency and water quality.^[^ ^16^ ^]^ Furthermore, lignin‐based materials hold considerable promise in biomedicine, with applications in tumor photothermal therapy and controlled drug delivery, illustrating their potential to contribute to targeted, minimally invasive treatments.^[^ ^17^, ^18^, ^19^ ^]^ Additional advances have been achieved in areas such as photothermal self‐healing, adsorption, and phase‐change systems, expanding the utility of lignin in functional applications.^[^ ^20^, ^21^, ^22^ ^]^ The evolution and development of lignin‐based photothermal materials, as shown in **Figure** **2**, demonstrate their emerging potential for sustainable applications.

However, despite these advancements, the practical implementation of lignin‐based photothermal materials faces several critical challenges. The structural complexity and inherent variability of lignin contribute to inconsistencies in material properties, which complicates extraction and purification processes.^[^ ^23^ ^]^ Furthermore, issues related to long‐term stability and scalability must be addressed to facilitate wider adoption.

Recent years witnessed substantial progress in understanding the chemical structure and properties of lignin, laying a solid foundation for exploring its photothermal conversion capabilities. However, systematic reviews dedicated to lignin‐based photothermal research remain scarce. Guan et al.^[^ ^24^ ^]^ investigated the application of lignin in photothermal materials, focusing on the relationship between the chemical structure of lignin and its derivatives and their photothermal conversion mechanisms. However, their exploration of lignin's potential applications in other fields is limited and lacks comprehensiveness.

This review offers a comprehensive analysis of lignin‐based photothermal materials, covering the underlying principles, classification, enhancement strategies, and current applications. By systematically comparing lignin with other conventional photothermal materials, the study highlights its advantages and limitations, providing a deeper understanding of its potential in this field and offering valuable insights for further development and optimization. It examines the structural characteristics of lignin, the mechanisms governing photothermal conversion, and the progress across various application domains while addressing challenges and future research directions. The review highlights the photothermal performance mechanisms of lignin, emphasizing its low cost, environmental sustainability, and efficient photothermal conversion ability. It summarizes strategies for improving photothermal efficiency and outlines promising applications in seawater desalination, photothermal therapy, energy storage, and other photothermal‐driven processes. This review highlights the main challenges and future prospects for advancing lignin‐based photothermal technologies. Through a systematic synthesis and analysis of existing studies, this review aims to furnish researchers with valuable insights and references, promoting further development and application of lignin‐based photothermal materials. Ultimately, this review aims to contribute to addressing energy crises and environmental challenges by advancing the practical application of lignin in sustainable photothermal technologies.

## 2 Lignin: A Natural Aromatic Polymer

### 2.1 Lignin Sources

**Figure 3:**
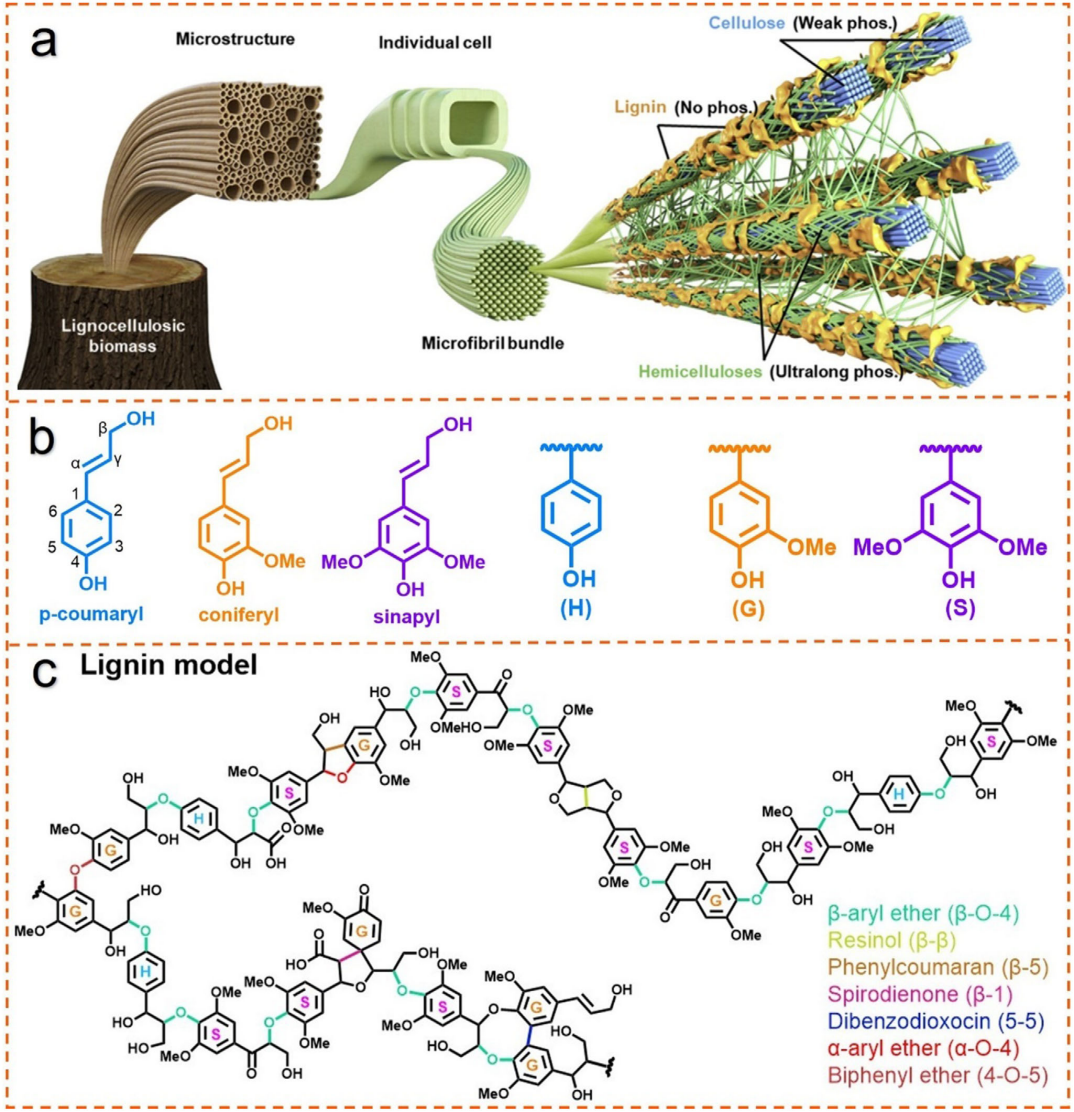
Fundamental structure and linkages of lignin. a) The composition of lignocellulose includes three main fractions: lignin, cellulose, and hemicellulose.^[^ ^52^ ^]^ Copyright 2022, Elsevier b) The three phenylpropanol monomers of lignin consist of p‐coumaryl, coniferyl, and sinapyl alcohols, as well as the corresponding p‐hydroxyphenyl (H), guaiacyl (G), and syringyl (S). c) Lignin structural units and major linkages.

Lignocellulose as the main component of the plant cell wall, which is responsible for the plant's support mechanisms.^[^ ^36^ ^]^ As shown in **Figure** **3**a, Lignin, cellulose, and hemicellulose are the most important lignocellulose components, which combine to make wood and other plant tissues to maintain a specific structure and strength.^[^ ^37^, ^38^, ^39^, ^40^ ^]^ Cellulose, a long‐chain polysaccharide composed of β‐1,4‐glycosidic bonded glucose units, ranks among the most abundant organic polymers in nature. As a major structural component of wood and plant cell walls, cellulose primarily imparts mechanical strength and stiffness to plants.^[^ ^41^ ^]^ Hemicellulose, a heterogeneous polysaccharide with a structure more complex than that of cellulose, comprises sugar units such as xylose, pentose, hexose, and deoxyhexose in various linkages. Functioning as a structural bridge between cellulose and lignin, hemicellulose enhances cell wall flexibility and contributes to its overall stability.^[^ ^42^ ^]^ Lignin, recognized as the predominant aromatic polymer in nature, accounts for ≈30% of the organic carbon content in the biosphere. Lignin is a crucial component of plant cell walls, primarily sourced from wood, the stems of herbaceous plants, secondary plant structures (such as bark and roots), and agricultural residues (including straw, corn stover, and sugarcane bagasse).^[^ ^43^ ^]^ Lignin is essential for the cell wall's toughness and rigidity, supporting water transport and offering protection against pathogens and environmental stresses.^[^ ^44^ ^]^ However, lignin presents notable challenges in industrial applications, particularly within the paper industry, where its presence adversely impacts paper whiteness and strength, posing a substantial hurdle that must be addressed to enhance product quality. Moreover, given its complex and heterogeneous structure, the efficient conversion of lignin into biofuels, chemicals, and high‐value materials has become a prominent research focus.

### 2.2 Lignin Structure

Lignin represents a structurally complex and heterogeneous natural macromolecule, primarily built from three phenylpropanoid monomers: p‐coumaryl, coniferyl, and sinapyl alcohols, commonly known as monolignols.^[^ ^45^ ^]^ These monolignols polymerize to form the structural units p‐hydroxyphenyl (H), guaiacyl (G), and syringyl (S) structural units, which integrate into the lignin polymer to create a highly branched and intricate architecture as illustrated in Figure 3b.^[^ ^46^, ^47^, ^48^ ^]^ Lignin substructures encompass various interunit linkages characterized by randomly connected C–C bonds (such as 5‐5, β‐5, β‐1, and β‐β) or C‐O bonds (including, α‐O‐4, 4‐O‐5, and β‐O‐4) at diverse locations through dehydration polymerization (Figure 3c).^[^ ^23^, ^49^ ^]^ Lignin's structural complexity is further enriched by functional groups along its side chains, such as methoxy, carboxyl, phenolic hydroxyl, and other carbonyl groups, which contribute to its diverse reactivity and biological functions.^[^ ^36^ ^]^ The β‐O‐4 ether linkage is the predominant interunit bond, accounting for more than 50% of lignin's linkages, a feature that influences both its stability and potential for depolymerization. Variation in the proportions of monolignols and linkage types is determined by the lignin source, resulting in distinct structural profiles across plant types.^[^ ^50^ ^]^ Coniferous lignin, for instance, consists primarily of guaiacyl units bonded through both ether and carbon‐carbon linkages, endowing it with a denser structure. In contrast, broadleaf lignin contains roughly equal amounts of guaiacyl and syringyl units, while herbaceous lignin incorporates all three units of H, G, and S. This variability in structural units also results in differences in cross‐linking and rigidity, endowing lignin with unique mechanical and chemical properties across plant families.^[^ ^46^, ^51^ ^]^

### 2.3 Lignin Classification

The extraction of lignin from lignocellulose represents a promising approach for the direct utilization of lignocellulosic biomass.^[^ ^38^ ^]^ However, it is worth noting that the structural and some physicochemical properties of extracted lignin are significantly influenced by both the extraction method employed and the original lignocellulosic source.^[^ ^23^ ^]^ As delineated in **Table** **1**, the main industrial extraction methods for lignin include kraft, soda, sulfite, and organic solvent methods.^[^ ^53^ ^]^ The Kraft pulping process, in particular, remains the most widely adopted technique for lignocellulose treatment, contributing to ≈85% of global lignin production.^[^ ^54^ ^]^ The traditional Kraft process involves the dissolution of lignin in a mixture of sodium hydroxide and sodium sulfide, commonly referred to as white liquor, under high temperature and high pH conditions to facilitate delignification.^[^ ^51^, ^53^ ^]^ Generally speaking, lignin derived through the sulfate process contains a small fraction of sulfur‐containing groups (1 to 3 wt%). Thus, commercial kraft lignin is often sulfonated to enhance its water solubility, a crucial step for large‐scale lignin utilization and its subsequent conversion into high‐value lignin‐based products.^[^ ^42^, ^51^ ^]^ The soda pulping process, by contrast, is primarily employed for the treatment of non‐wood lignocellulosic materials such as grasses, bagasse, and straw.^[^ ^8^ ^]^ Unlike the Kraft process, no sulfur is introduced during lignin dissolution, which offers significant potential for generating high‐value lignin derivatives due to the absence of sulfur.^[^ ^38^, ^51^ ^]^ The sulfite method is another widely used approach in the pulp and paper industry, which mainly involves the reaction of lignin with metal bisulfites and sulfur dioxide (calcium, magnesium, or sodium as counter ions). Compared to the Kraft process, the lignosulfonates extracted by this process have higher molecular weights due to the combination of sulfonic acid groups with the framework of lignin.^[^ ^55^ ^]^ The organic solvent method mainly utilizes organic solvents as biomass delignification agents to dissolve lignin and hemicellulose, thereby facilitating lignin removal.^[^ ^53^ ^]^ The technique presents a promising alternative to conventional pulping technologies, owing to its environmentally friendly preparation process and high purity, free from sulfur and ash contaminants.^[^ ^9^, ^56^ ^]^

**Table 1:** Summary of industrial lignins obtained by different extraction methods and their properties.

Lignin type	Structure	M_w_ [10^3^ gmol^−1^]	Solubility	Extraction method	Sulphur [%]	*T_g_* [°C]	Refs.
Kraft lignin	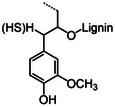	1.5 to 5 (up to 25)	Alkali; some organic solvents (DMF, pyridine and DMSO)	Kraft process using a mixture of NaOH and Na_2_S	1.0 to 3.0	124–174	[9, 38, 51, 55]
Soda lignin	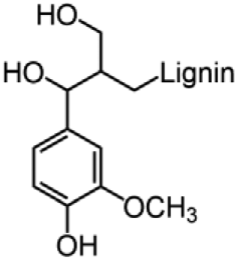	1–50 (up to 150)	Alkali	Soda process using 13–16% of NaOH	0	130–168	[9, 51, 55]
Lignosulfonate	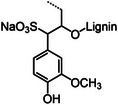	0.8–3 (up to 15)	Water	Sulfite process using hydrogen sulfite and sulfur dioxide	3.5 to 8.0	≈130	[9, 51, 55]
Organosolv lignin	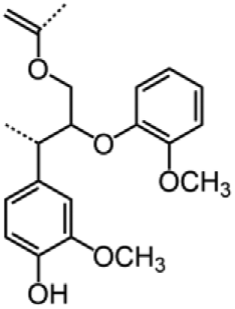	0.5–5	Wide range of organic solvents	Organosolv process using acetic acid ethanol, methanol, etc. mixed with water	0	90–112	[9, 51, 55]

In addition to traditional pulping processes, significant progress has been made in lignin refining technologies, including steam explosion, acid hydrolysis, pyrolysis, ionic liquid dissolution, and deep eutectic solvent dissolution.^[^ ^57^, ^58^ ^]^ Steam explosion involves the treatment of biomass with high‐temperature, high‐pressure steam, followed by rapid depressurization, which causes steam expansion and disrupts the fiber structure. This process is efficient and cost‐effective, but is typically used as a pretreatment step in combination with other processes, and the lignin produced tends to exhibit higher heterogeneity.^[^ ^59^, ^60^ ^]^ Hydrolyzed lignin, a byproduct of cellulose hydrolysis for bioethanol production, is denser and more cross‐linked than sulfate lignin, making depolymerization more challenging.^[^ ^61^ ^]^ Pyrolysis, conducted under anoxic conditions, converts lignocellulosic biomass into bio‐oil, char, and gases (H₂, CO, CO₂, CH₄), with lignin mainly degrading into phenolic compounds and char. Ionic liquids (ILs) show promise in ionic solvent methods, however, their high cost limits industrial applications.^[^ ^62^ ^]^ Deep eutectic solvents (DESs), composed of hydrogen‐bond donor‐acceptor pairs in specific ratios, offer advantages such as low cost, biodegradability, and ease of preparation. DESs are considered an environmentally friendly and efficient medium for lignin extraction, demonstrating high selectivity and low waste generation in lignin separation, and can be recycled.^[^ ^63^ ^]^

## 3 Photothermal Conversion of Lignin

Solar photothermal conversion, recognized as an efficient approach for converting solar energy into thermal energy, have attracted considerable interest from both the research community and industry, particularly in light of the escalating global energy demand and growing environmental awareness.^[^ ^6^, ^64^ ^]^ Lignin, as a natural and renewable polymer material, offers a more abundant and environmentally friendly source of raw materials compared to traditional photothermal materials such as precious metals and semiconductor materials, making it suitable for large‐scale applications. Additionally, the biocompatibility and biodegradability of lignin provide promising prospects for its use in fields such as medicine and environmental protection.^[^ ^36^ ^]^ As shown in **Table** **2**, compared to some traditional photothermal materials, its photothermal conversion efficiency remains insufficient, which may limit its widespread use in certain high‐demand applications. Therefore, the photothermal performance of lignin will require further optimization and modification in the future to address these limitations.

**Table 2:** Comparative analysis of photothermal properties of lignin and traditional materials.

Photothermal materials	Material sources	Photothermal mechanism	Photothermal efficiency	Environmental Friendliness	Material cost	Resource Sustainability	Thermal stability	Mechanical strength	Refs.
Noble metal	Au, Ag, Pd, Al, Cu	plasmonic localized heating	high	moderate	high	poor (resource scarcity)	high	high	[80, 81, 82, 83, 84]
Semiconductors	TiO_2_, CuS	nonradiative relaxation	high	moderate	medium	moderate (limited mineral resources)	moderate to high	high	[6, 85, 86]
Carbon‐based materials	graphene, graphite	thermal vibrations of molecules	medium	medium	high	medium (from fossil resources)	medium	high	[6, 87, 88]
Organic Polymer	polyaniline, polypyrrole	thermal vibrations of molecules	high	moderate	medium	moderate (based on petrochemical)	medium	low	[6, 77, 89]
Lignin	wood processing waste	thermal vibrations of molecules	Medium	excellent (natural, environmentally friendly, biodegradable)	very low	excellent (abundant, renewable, and sustainable sources)	medium	moderate	[12, 16, 35]

### 3.1 Basic Conversion Mechanisms of Photothermal Materials

**Figure 4:**
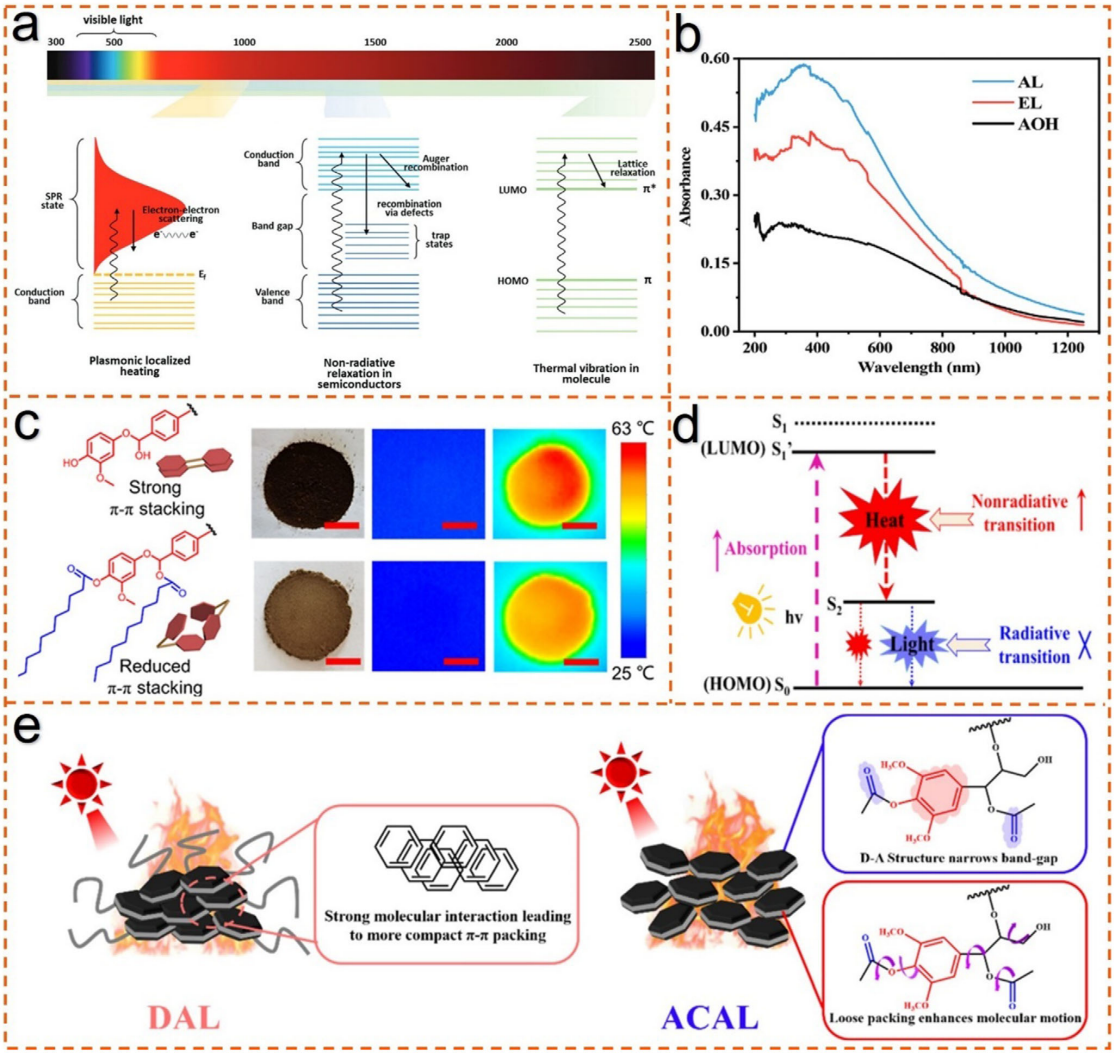
Mechanism of photothermal properties in lignin. a) Photothermal effects stem from various mechanisms.^[^ ^7^ ^]^ Copyright 2019, Royal Society of Chemistry. b) UV–Vis–NIR spectra of AL, EL, and AOH.^[^ ^14^ ^]^ Copyright 2022, Royal Society of Chemistry. c) Photothermal comparison between lignin and long chain modified lignin.^[^ ^12^ ^]^ Copyright 2021, American Chemical Society. d) Non‐relaxation radiation mechanism of lignin.^[^ ^78^ ^]^ Copyright 2022, Royal Society of Chemistry. e) Photothermal comparison between lignin and electron‐withdrawing group lignin.^[^ ^35^ ^]^ Copyright 2022, Royal Society of Chemistry.

The conversion efficiency of photothermal materials is not only affected by the light absorption coefficient of the material, but also closely related to the structural composition, morphology, and surrounding environment.^[^ ^65^ ^]^ In this section, as shown in **Figure** **4**a, three basic mechanisms involved in the photothermal conversion process are briefly outlined.^[^ ^7^ ^]^ 1) Plasmonic localized heating. Metal nanostructures including gold, silver, and platinum contain a large number of highly concentrated free and polarizable electrons inside and these plasmonic nanoparticles can efficiently absorb the energy of incident photons through electronic transitions under illuminating conditions.^[^ ^66^ ^]^ Notably, localized surface plasmon resonance (LSPR) is observed when free electrons inside the metal are appropriately illuminated, which can be further enhanced when the photon energies match the relevant energy bands of the LSPR. The LSPR is mainly presented in three forms: near‐field enhancement, thermoelectric generation, and photo‐thermal conversion, all of which contribute to enhanced light absorption and local field amplification.^[^ ^6^, ^65^, ^67^ ^]^ 2) Non‐radiative relaxation of semiconductors. By capturing photon energy that surpasses the bandgap, semiconductor materials are capable of converting solar energy into thermal heat. Electrons in the valence band (VB) can be excited to the conduction band (CB), followed by the generation of excited‐state electrons and holes in the CB and VB, respectively.^[^ ^68^, ^69^ ^]^ Subsequently, the excited‐state electrons and holes relax to the respective edges of the CB and VB, and ultimately convert solar energy into heat. In semiconductors, thermal losses will increase when charge carriers that release phonons instead of photons recombine, which leads to a localized temperature increase in the lattice.^[^ ^70^ ^]^ The photothermal performance loss in direct bandgap materials is largely attributed to the release of photons by radiative recombination of excited carriers (electrons and holes). Additionally, the reflection of light with wavelengths shorter than the band edge will also result in lower photothermal conversion efficiency.^[^ ^6^, ^64^ ^]^ 3) Thermal vibration in molecules. Carbon‐based materials and polymers also have excellent light absorption characteristics and can generate heat through lattice vibrations. The energy difference between the σ and σ* orbitals of most single‐bond carbons (e.g., C–C, C–O, and C–H) is substantial, making it difficult to excite.^[^ ^71^ ^]^ Moreover, conjugated π bonds can induce a red shift in the absorption spectrum. When a large number of π bonds are present, the energy level difference between the highest occupied molecular orbital (HOMO) and the lowest unoccupied molecular orbital (LUMO) decreases, resulting in a red shift in the absorption spectrum.^[^ ^68^ ^]^ Additionally, conjugated π bonds induce a red shift in the absorption spectrum. With an increased presence of π bonds, the energy gap between the HOMO and the LUMO narrows, leading to a red shift in the absorption spectrum.^[^ ^64^ ^]^ Thus, during electronic transitions, π electrons are excited from the HOMO to the LUMO, and the excited electrons subsequently return to the HOMO energy level, releasing heat when the energy of the incident photon matches the energy required for these electronic transitions within the organic molecule.^[^ ^6^, ^72^, ^73^ ^]^

### 3.2 Lignin Photothermal Conversion Mechanism

In industrial processes, the natural structure of lignin degrades under acidic, alkaline, and high‐temperature conditions, leading to the formation of chromophores (such as carbon‐carbon double bonds, aromatic rings, quinone methyl, and quinone conjugates) and co‐chromophores (such as phenolic hydroxyl groups).^[^ ^74^ ^]^ To further investigate the photothermal conversion mechanisms of lignin, exploring the relationship between its band gap structure and molecular interactions with photon radiation is essential.^[^ ^35^, ^75^ ^]^ The photothermal effect of lignin primarily depends on the chemical structure and *π–π* interactions within the molecular framework.^[^ ^12^ ^]^ The extensive aromatic ring structure in lignin promotes efficient photon absorption in the visible and near‐infrared (NIR) spectrum.^[^ ^19^ ^]^ Upon illumination, aromatic rings undergo photoexcitation, leading to electronic transitions that rapidly convert absorbed energy into heat, subsequently releasing heat into the surrounding medium.^[^ ^14^ ^]^ Therefore, the photothermal conversion mechanism of lignin primarily involves two key processes: light absorption and non‐radiative relaxation. Due to its rich aromatic structure and the presence of functional groups such as hydroxyl and methoxy, lignin can effectively absorb light at specific wavelengths, typically in the ultraviolet and visible light ranges.^[^ ^6^, ^14^, ^19^ ^]^ The free electrons in these structures can easily be excited from the π orbitals to the π* orbitals under low‐energy radiation.^[^ ^14^, ^76^ ^]^ When the energy of incident photons matches the electronic transition of lignin, π electrons are excited from the ground state to a higher energy level.^[^ ^16^ ^]^ These π electrons return to the ground state through vibrational‐electronic coupling and release excess energy in the form of heat. Furthermore, the conjugation or hyperconjugation of the π orbitals can alter the electronic transition between the ground and excited states.^[^ ^77^ ^]^ As the number of π bonds increases, the band gap energy decreases, reducing the energy required for electrons to transition from the low‐energy state to the high‐energy state. The shift in energy levels plays a crucial role in the photothermal conversion efficiency.^[^ ^6^, ^35^ ^]^ Alternatively, non‐radiative relaxation represents another crucial process. Following light absorption, lignin molecules undergo non‐radiative relaxation, transitioning from an excited state back to the ground state, thereby releasing thermal energy into the surrounding environment.^[^ ^65^, ^78^ ^]^ Thermal energy can be efficiently transferred within the material due to the excellent thermal stability and thermal conductivity of lignin given by the chemical structure.^[^ ^79^ ^]^ However, some light energy continues to be lost through radiative transitions. Therefore, suppressing radiative transitions serves as a key approach to improving photothermal conversion efficiency.

The structure of lignin plays a crucial role in determining its photothermal conversion efficiency. Li et al.^[^ ^14^ ^]^ compared the photothermal conversion efficiencies of three types of lignin: alkaline lignin (AL), low molecular weight alkaline lignin (AOH), and enzymatic hydrolysis lignin (EL). As shown in Figure 4b, UV–Vis–NIR analysis showed a characteristic benzene ring absorption peak at 356 nm for all lignin and maintained high absorbance at 808 nm (AL: 0.18, EL: 0.16, AOH: 0.11). Despite AL having the highest absorbance at 808 nm, AOH exhibited the best photothermal conversion efficiency (η = 53.7%), highlighting that molecular structure, not just light absorption, is key to photothermal performance. The temperature rise followed the order AOH > AL > EL, which contrasts with their molecular weight trend (AL > EL > AOH). AOH's higher hydroxyl content, intermolecular hydrogen bonding, and *π–π* stacking enhanced non‐radiative energy release, improving photothermal conversion. Zhao et al.^[^ ^5^ ^]^ treated lignin with demethylation, which increased the phenolic hydroxyl content in lignin, thereby promoting intermolecular interactions, particularly *π–π* stacking. The demethylation process altered the molecular structure of lignin, and after the removal of methyl groups, the aggregation of lignin molecules became more compact, further facilitating the delocalization of electrons and significantly enhancing its photothermal conversion ability. Therefore, these results demonstrate that the photothermal conversion efficiency of lignin is closely linked to its light absorption capacity and is also significantly influenced by its molecular structure. Similarly, modifying the structure of lignin also affects its photothermal behavior.

Further elucidation of the photo‐thermal mechanism of lignin has been provided by other researchers. As depicted in Figure 4c, photothermal experiments were performed to compare the inhibitory effect of long alkyl chain‐modified lignin on molecular *π–π* stacking. In addition, the photothermal effect of lignin stemming from the non‐radiative relaxation of *π–π* stacking was verified by density functional theory calculations. The photothermal conversion process mainly comprises two stages: light energy absorption and heat generation. The energy gap between LUMO and HOMO of lignin materials is lower than that of other materials due to the presence of *π–π* stacking structure. Excited electrons can transition from the LUMO state to the HOMO state generating heat and accompanied by a nonradiative transition when light strikes lignin materials (Figure 4d).^[^ ^78^ ^]^ Lei et al.^[^ ^35^ ^]^ proposed an approach to enhance the photothermal effect of lignin by incorporating electron‐withdrawing groups, as illustrated in Figure 4e. By introducing electron‐withdrawing acetyl groups, a donor–acceptor (D–A) structure was successfully constructed within the lignin molecule, thereby narrowing the band gap of the modified lignin (ACAL) and enhancing its light absorption capability. Moreover, the reduction in hydrogen bonding in ACAL, due to decreased hydroxyl content, weakens intermolecular interactions and enhances molecular mobility, which further promotes the non‐radiative decay of lignin and ultimately boosts photothermal conversion.

In summary, the photothermal conversion capability of lignin can be attributed to the vibrational activity of its aromatic rings and other structural elements, which effectively convert light energy into thermal energy through non‐radiative relaxation. Lignin holds significant potential for photothermal conversion, offering promising prospects for the advancement of sustainable energy technologies.

### 3.3 Strategies to Enhance the Capacity of Lignin‐Based Materials for Photothermal Conversion

The presence of various reactive groups, including hydroxyl and methoxy groups, in lignin's chemical structure allows for chemical modification to further enhance its photothermal properties and expand its application scope.^[^ ^13^ ^]^ Furthermore, lignin‐based photothermal materials offer a cost‐effective alternative to noble metal nanoparticles, significantly reducing the economic burden associated with photothermal technologies. This section provides a detailed discussion of the advantages of lignin in the field of photothermal materials and its preparation strategies. Strategies for enhancing lignin's photothermal efficiency include. 1) Nanoscale processing. processing lignin into nanoparticles through nanotechnology can significantly increase its specific surface area and further enhance photothermal conversion efficiency. Ma et al.^[^ ^34^ ^]^ successfully fabricated a robust and photothermal superhydrophobic coating using dual‐size lignin micro‐nanospheres (LMNSs), which include lignin microspheres (m‐LMNSs) and nanospheres (n‐LMNSs). The micro‐nanospheres settled sequentially on the substrate surface due to gravitational forces. The photothermal effect of the coating results in a rapid surface temperature rise from ≈13 to 112 °C within 60 s under laser irradiation, demonstrating excellent photothermal responsiveness. The innovative structural regulation approach greatly improves the durability and photothermal performance of lignin‐based superhydrophobic coatings, confirming the method's feasibility for practical applications. 2) Chemical modification. The light absorption ability and thermal stability of lignin can be further improved by introducing specific light‐absorbing groups or combining with other materials with excellent photothermal properties. Lei et al.^[^ ^35^ ^]^ modified lignin through acetylation, with the introduction of acetyl groups forming electron D‐A structures that enhanced light absorption. Under 808 nm laser irradiation at 0.51 W cm⁻^2^, the photothermal conversion efficiency of acetylated lignin (ACAL) reached 73.2%, 37% increase compared to unmodified lignin, demonstrating that chemical modification significantly improves the photothermal effect of lignin materials. 3) Preparation of composite materials. Combining lignin with materials such as graphene, porous carbon materials, and metal materials facilitates the formation of composite photothermal materials. Shao et al.^[^ ^90^ ^]^ prepared a lignin‐guided solution of copper sulfide nanoparticles. The PVA photothermal film fabricated using this lignin‐guided CuS stabilization solution exhibited excellent solar absorption capacity (about 95%) and good uniform dispersion of CuS nanoparticles. The film demonstrated a high photothermal conversion efficiency (≈49.43%), providing a novel approach for synthesizing metal nanoparticles regulated by lignin and for utilizing lignin as a multifunctional photothermal material in energy production and environmental remediation.

Lignin, as a photothermal material, holds significant potential due to its natural abundance, eco‐friendliness, good biocompatibility, and ease of chemical modification. Enhancing its photothermal conversion efficiency through various strategies can expand its applications in the photothermal field. However, the feasibility and limitations of each approach need further consideration. For instance, nanoscale processing can be expensive, while chemical modification necessitates specific reaction conditions. Therefore, it is crucial to select the most appropriate strategy based on the specific context in order to maximize the practical applications of lignin.

## 4 Applications of the Lignin Photothermal Effect

The application fields of lignin photothermal materials are gradually broadening driven by an increasingly sophisticated understanding of their photothermal conversion mechanisms. As a biopolymer with high natural abundance and inherent solar‐thermal conversion properties, lignin offers a promising solution to the growing global demand for renewable and sustainable energy sources. For example, in the field of seawater desalination, lignin‐derived photothermal materials are being explored for the development of high‐performance evaporators, thereby significantly enhancing desalination efficiency.^[^ ^91^ ^]^ In the area of photothermal response materials, lignin‐based substances are being utilized to create materials with precisely tunable optical properties, presenting distinct advantages for the design of dynamic and adaptive systems.^[^ ^92^ ^]^ Furthermore, the application of lignin photothermal materials is proposed to extend into a wide array of domains, including biomedicine, phase‐change energy storage, and other cutting‐edge technologies.^[^ ^19^, ^79^, ^93^ ^]^ This review provides a comprehensive synthesis of recent advancements in lignin photothermal nanomaterials, highlighting key case studies that underscore the transformative potential of these materials in addressing pressing challenges across a broad spectrum of scientific and industrial fields.

### 4.1 Photothermal Responsive Materials

The photothermal conversion of light‐absorbing materials leads to the fundamental mechanical deformation of thermal‐responsive devices, such as bending, twisting, rotating, and jumping actions.^[^ ^25^ ^]^ In the design of photothermal actuators, several factors must be carefully considered, including the degree of photothermal conversion, the resulting stresses and strains, material durability, and the response time. Research into photothermal‐responsive materials is not only critical for understanding the underlying response mechanisms of these materials but also holds significant practical implications for the development of advanced optoelectronic devices, smart materials, and other emerging technologies. Shape memory polymers (SMPs) are a class of polymers with the unique ability to memorize various shapes and recover a pre‐determined form when exposed to specific stimuli, such as heat, magnetic fields, electric fields, moisture, or light.^[^ ^94^ ^]^ Li et al.^[^ ^95^ ^]^ successfully synthesized a lignin‐copoly (ester‐amine) elastomer with shape memory properties using a one‐pot, two‐step condensation reaction. Thermal‐responsive shape memory copolymer elastomer demonstrated enhanced mechanical and thermal properties due to the interaction between lignin and other polymers. Additionally, the incorporation of lignin enabled the copolymer to exhibit significant shape memory effects, allowing it to revert to its original shape upon appropriate thermal stimulation. As research into thermal‐responsive materials advances, significant attention is being directed toward achieving more precise and controllable temperature‐responsive behaviors, which is crucial for expanding their practical applications. Lignin, as a clean and abundant energy source, has emerged as a critical factor in advancing the development of sustainable technologies.

**Figure 5:**
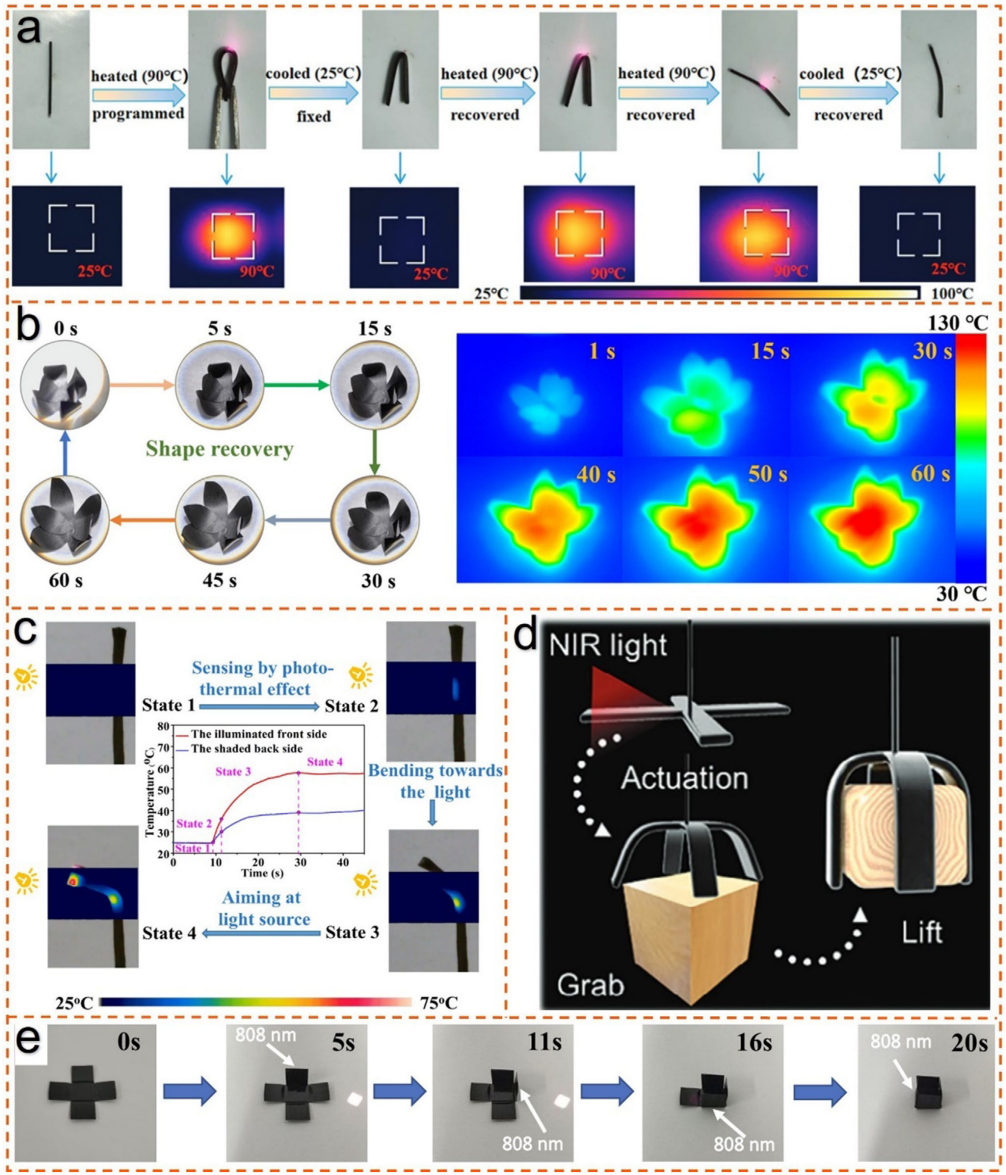
Photothermal properties and actuation of lignin‐based photoresponsive elastomers. a) Shape memory effect observed under NIR light at 808 nm with a power density of 0.47 W cm^−2^, where the distance between the sample and light source was 10 cm.^[^ ^14^ ^]^ Copyright 2022, Royal Society of Chemistry. b) The shape memory and thermographic behavior of ELID30 under 3 sun radiation conditions.^[^ ^97^ ^]^ Copyright 2022, Elsevier c) Temperature variations on the illuminated side (front) and the shaded side (back) of P100L6Z15@600% during the 808 nm near‐infrared laser tracking process at a power density of 0.95 Wcm^−^^2^.^[^ ^78^ ^]^ Copyright 2022, Royal Society of Chemistry. d) Schematic soft gripper sorting systems enabled by A_2_L BPTE actuators.^[^ ^96^ ^]^ Copyright 2022, Wiley. e) Schematic diagram of self‐folding for LP4‐50.^[^ ^25^ ^]^ Copyright 2022, American Chemical Society.

Biomass lignin has been shown to be an efficient photothermal agent for the preparation of multifunctional smart elastomer composites, primarily attributed to the its conjugated structure, which effectively promotes the jump of electrons from low‐energy orbitals to high‐energy states.^[^ ^14^, ^96^ ^]^ Notably, NIR light absorbed by lignin is predominantly released through non‐radiative decay processes. Li et al.^[^ ^14^ ^]^ directly incorporated AL as a functional agent into polyethylene elastomer (POE) through a solvent‐free melt compounding process to prepare multifunctional smart elastomer composites. Under near‐infrared light stimulation, the elastomer composites exhibit a light‐triggered shape memory effect. **Figure** **5**a visually demonstrates the shape memory effect of P32L8. When locally heated to 90 °C by NIR radiation, the material is first programmed from a flat permanent shape into a temporary bent “V” shape, then quickly cooled to room temperature. The temporary “V” shape is fixed after the external force is removed, and this temporary shape remains stable at room temperature. The sample can respond to near‐infrared light within 30 s, rapidly returning from the temporary “V” shape to its original linear form. In similar study, Jin et al.^[^ ^97^ ^]^ utilized enzymatic hydrolysis lignin, itaconic acid, and 1,12‐dodecanediol as raw materials to fabricate lignin‐based light‐driven shape‐memory polymers, without any chemical modification of the lignin. ELID30, a composite material from this study, demonstrated the ability to reach temperatures above its glass transition temperature (*T_g_*) under solar radiation, promoting the light‐induced shape memory effect. A temporary shape was programmed into the form of a black rose, which, upon exposure to solar radiation, transitioned significantly from a closed bud to an open flower (Figure 5b). The restoration of each petal's specific shape was dependent on the angle of irradiation. Building upon the fundamental principles of photothermal shape‐memory materials, the potential for dynamic movement can be further investigated. Through careful design and development, photothermal‐responsive materials exhibit sunflower‐like phototropic behavior. Upon illumination, these materials undergo shape changes and spontaneously adjust their orientation toward the light source. Tu et al.^[^ ^78^ ^]^ proposed a construction method known as the photothermal domino strategy, which sequentially optimizes photothermal generation, thermal conduction, and thermally driven responses through coordination effects. This approach enables the development of fast‐responsive biomimetic phototropic materials with a wide temperature‐responsive range. The photothermal conversion efficiency of lignin was enhanced 2.3‐fold through Zn^2^⁺ – π coordination interactions, which reduced the energy bandgap between the HOMO and LUMO of lignin molecules and promoted the non‐radiative transition of lignin free radicals. By grafting lignin‐based coordination compounds onto an EPDM matrix, the interface coordination bonds effectively enhanced the thermal conductivity between lignin and the polymer matrix. Furthermore, mechanical training promoted the alignment of polymer chains at the interface, reducing entropy within the lignin–EPDM elastomer composite and accelerating the material's thermal/photothermal reversible driving performance. The composite also exhibited a wide temperature‐responsive window (30–90 °C). Similar to natural sunflowers, the lignin–EPDM elastomer composite exhibited phototropic motion due to asymmetric deformation caused by the temperature difference between the irradiated front and back surfaces (Figure 5c). The light‐tracking process was completed in just 2.5 s, achieving a speed that surpassed that of real sunflowers and previous artificial phototropic materials. This adaptive response improves the material's intelligent responsiveness, thereby creating new opportunities for applications in autonomous adjustment, solar energy harvesting, and related fields.

With the growing demand for environmental protection and sustainable development, research on photothermal‐responsive materials has gradually shifted from traditional petroleum‐based raw materials to greener, more economical, and environmentally friendly biomass‐based alternatives. The production of petroleum‐based materials depends on limited fossil resources and contributes to environmental pollution. In contrast, biomass‐based materials, as natural and renewable resources, offer significant potential for reducing carbon emissions and promoting sustainable resource utilization. With advances in green chemistry technologies, the synthesis and application of fully biomass‐derived materials continue to make breakthroughs, contributing positively to the promotion of the green economy and sustainable development. Sun et al.^[^ ^96^ ^]^ introduced a fully biomass‐derived photothermal elastomer (BPTE), which was prepared using a simple, chemical‐free method with biomass‐derived lignin, lipoic acid, and phytic acid as raw materials. The study demonstrated that phenolic groups in AL reacted with thiol radicals at the chain ends of PLA, effectively preventing PLA network depolymerization at room temperature and enabling a dynamic covalent disulfide‐crosslinked polymer network. By adjusting the content of AL, the microstructure of the polymer network could be tuned, resulting in high mechanical strength, excellent ductility, rapid self‐healing, hydrophobicity, anti‐swelling properties, and recyclability. Additionally, the *π–π* conjugated structure in AL contributes to enhancing the photothermal conversion efficiency, giving the material excellent photothermal responsiveness under visible and near‐infrared light. For instance, Figure 5d shows the development of a near‐infrared‐driven four‐arm soft actuator, a study that not only advanced the high‐value utilization of AL but also offered an effective solution to the performance balance and sustainability challenges in the field of photothermal materials. Chen et al.^[^ ^25^ ^]^ provided new insights into the development of high‐performance and green lignin‐based photothermal agents. Lignin‐based photothermal actuator has been developed to demonstrate rapid light‐driven contraction capabilities. Light‐driven shrinkage of up to 18% is achieved when lignin is blended with a polyamide elastomer derived from castor oil. Stress‐induced strain energy under load is firmly locked due to the crystals in the polymer matrix acting as switchable segments. The strain energy is rapidly released by the photothermal process, resulting in a pronounced contraction. The composite material contracts in multiple directions and exhibits dynamic bending when locally irradiated by NIR 808 nm laser. Additionally, as shown in Figure 5e, the LP4‐50 is shaped into a flat box, which, under NIR laser irradiation, returns to its 3D shape in just 20 s. It provides important insights for the development of lignin‐based photothermal responsive materials with high potential applications.

These findings highlight the vast potential of lignin‐based elastomer composites for precision remote‐controlled smart materials, with applications spanning robotics, machines, sensors, sterilization, and self‐repairing devices.^[^ ^98^ ^]^ Such advancements not only enhance the practical utilization of lignin but also contribute to the sustainable development and high‐value applications of biomass‐derived resources. The continued research on lignin‐based photothermal actuators and other non‐contact manipulation tools to realize faster response time, higher energy conversion efficiency and greater mechanical robustness, ultimately advancing the development of smart autonomous systems.

### 4.2 Photothermal Self‐Healing Materials

Under light exposure, photothermal self‐healing materials repair themselves by utilizing the heat generated. Lignin effectively absorbs light energy and converts it into heat, enhancing the self‐healing ability of the material. Its inclusion provides cost efficiency, environmental friendliness, and improved thermal stability, accelerating the repair process in damaged areas and thus extending the material's lifespan and performance.

**Figure 6:**
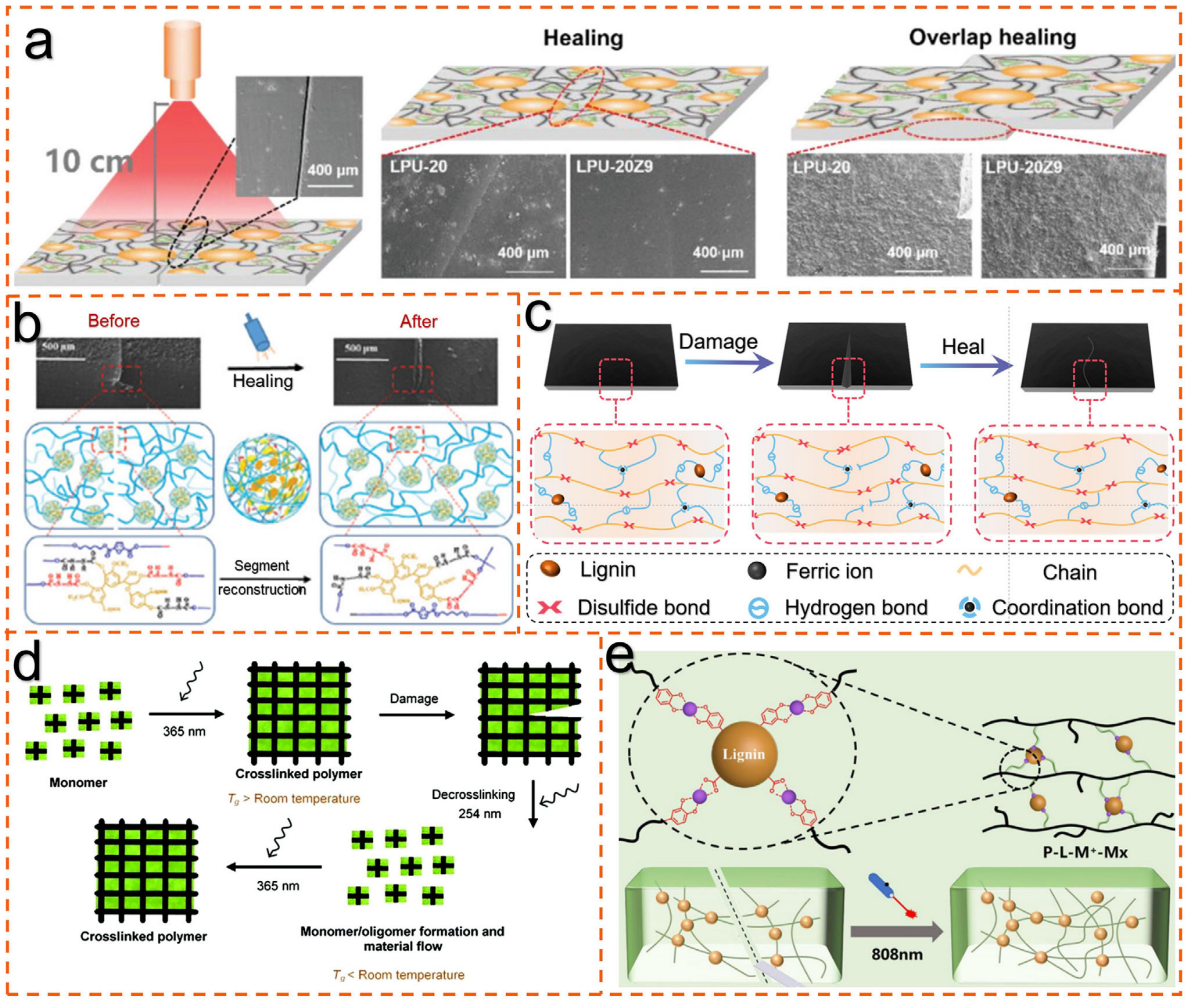
Lignin‐based photothermal self‐healing materials. a) High‐performance, light‐stimulation healable, and recyclable lignin‐based covalent adaptable networks.^[^ ^101^ ^]^ Copyright 2022, Wiley. b) Light‐triggered self‐healing phenomenon of sample, with the NIR laser power of 0.9 W cm^−2^.^[^ ^99^ ^]^ Copyright 2024, Wiley. c) Schematic diagram of the self‐healing mechanism of PL40 elastomers.^[^ ^15^ ^]^ Copyright 2022, Elsevier d) Formation of photo‐responsive polymer and self‐healing mechanism.^[^ ^100^ ^]^ Copyright 2021, Royal Society of Chemistry. e) Nickel‐catalyzed synthesis of self‐healing polyolefin composites: copolymerization with lignin cluster monomers and NIR‐triggered healing.^[^ ^22^ ^]^ Copyright 2024, Wiley.

Recent reports indicate that the lignin within the material absorbs light, triggering the release of thermal energy, causing the self‐healing agent to melt at elevated temperatures and uniformly flow out to fill and repair the damaged area (**Figure** **6**a). This process enhances the material's lifespan and durability while reducing reliance on traditional repair methods, thereby minimizing resource waste. Additionally, lignin, being derived from biomass, is environmentally friendly and aligns with sustainable development principles. Sun et al.^[^ ^99^ ^]^ successfully developed a novel multifunctional lignin‐modified polyester elastomer (LFPEe), which was prepared by combining 2,5‐furandicarboxylic acid (FDCA)‐based polyester oligomers (PPeF) with lignin (AOH). In the design of the material, particular attention was paid to the construction of dynamic bonds, especially hydrogen bonds and covalent carbonate ester bonds, which together form a self‐healing system. The covalent carbonate ester bonds exhibited distinct dynamic properties at a high temperature of 150 °C, manifested as stress relaxation, confirming their ability to self‐heal at elevated temperatures (Figure 6b). This excellent combination of photothermal functionality and dynamic covalent bonds imparts outstanding light‐triggered self‐healing performance to the LFPEe material. Sun et al.^[^ ^15^ ^]^ designed a multifunctional elastomer based on lignin. The polymer elastomer network is composed of ring‐opening polymerized poly(lipoic acid) (PLA), with the carboxyl groups of PLA forming coordination bonds and hydrogen bonds with Fe^3^⁺ and lignin. Lignin plays a crucial role in the photothermal effect, mechanical properties, and thermally induced self‐healing ability. To simulate practical damage, the elastomer was scratched, and under NIR laser irradiation, the elastomer exhibited the anticipated self‐healing behavior (Figure 6c). Increasing the temperature accelerated the flow of polymer chains, promoting the formation of the polymer network near the damaged area, thereby facilitating the self‐healing process. Lignin plays a key role in this process by absorbing NIR light and converting it into heat, causing the temperature near the damaged area to rise rapidly above 90.2 °C. This elevated temperature promotes polymer flow and contact at the damage edges, while also assisting iron ions in recombining with the PLA carboxyl groups. The damaged elastomer was able to essentially complete the self‐healing process. Tensile tests further revealed that untreated damaged elastomers failed rapidly under tensile stress, whereas the repaired samples, after NIR light and thermal treatment, exhibited tensile properties nearly restored to their original state. Specifically, the tensile strength of the self‐healing elastomer recovered 78.8% after 15 min of local heating, while the mechanical strength reached 98.2% after just 5 min of near‐infrared laser irradiation. This study provides a new perspective on the high‐value utilization of lignin.

UV‐responsive self‐healing polymers must satisfy the reversibility of UV‐active double bonds. When damage occurs, exposure to UV light is sufficient to induce bond cleavage without the need for external intervention. The material flows to the damaged area and repairs the crack. Once the damage is sealed, the crosslinked structure reforms under different UV wavelengths (Figure 6d).^[^ ^100^ ^]^ However, for the crosslinked state, the *T_g_* must be higher than room temperature to ensure the material remains stable during use. Therefore, the polymer must be designed with reversible UV‐active groups and maintain stability at room temperature. Introducing efficient photothermal conversion agents into polymers with low‐temperature molecular chain flexibility could enable rapid, real‐time self‐healing in various environments (e.g., low temperatures, underwater, seawater, organic solvents). Notably, lignin is a cost‐effective and sustainable photothermal conversion agent. Its abundant functional groups provide a foundation for the construction of dynamic bonds. However, the incompatibility between lignin and the polymer often results in poor photothermal efficiency and material performance. In situ polymerization in the presence of lignin offers a promising strategy to address this challenge. Chen et al.^[^ ^22^ ^]^ developed a lignin cluster polymerization strategy (LCPS) for the preparation of lignin‐polyolefin composite materials with real‐time self‐healing capabilities, exhibiting excellent self‐healing performance in various extreme environments. Initially, some polar functionalized olefin comonomers coordinate with metal‐ion‐modified lignin surfaces to form ion clusters. These comonomers then copolymerize with ethylene under the catalysis of α‐diimine nickel, generating functionalized polyolefin composites with uniformly distributed lignin. The formation of these clusters prevents catalyst poisoning from polar comonomers or lignin's polar groups, promoting efficient copolymerization of the polyolefin composite materials. The polyolefin matrix exhibits excellent chemical resistance and a low *T_g_*, enabling it to withstand complex environments such as polar solvents, seawater, and low temperatures. More importantly, this strategy ensures uniform dispersion of lignin, enhancing photothermal conversion efficiency and thermal conductivity at the lignin‐polymer interface, thereby accelerating the self‐healing process.

In summary, lignin‐based photothermal self‐healing materials, with their excellent photothermal conversion properties and self‐healing capabilities, demonstrate immense potential in smart materials and self‐healing systems. As research continues to advance, the optimization of the processing techniques and performance of lignin‐based photothermal self‐healing materials will further promote their application in fields such as electronic devices, building materials, and wearable technology, opening up broader application prospects and providing new solutions for sustainable development in smart materials.

### 4.3 Seawater Desalination

Seawater comprises ≈97% of Earth's surface water, highlighting the pressing need for the development of desalination technologies to alleviate freshwater scarcity.^[^ ^102^ ^]^ Among the array of alternative energy sources, solar energy, as a clean and renewable energy source capable of reducing reliance on conventional energy systems, simultaneously mitigating carbon emissions and advancing the goal of carbon neutrality.^[^ ^103^, ^104^ ^]^ Lignin‐based photothermal materials, with their abundant availability, low cost, and environmental sustainability, have become promising candidates for efficiently converting solar energy into thermal energy. In seawater desalination systems, lignin‐based photothermal materials are typically used as heat‐absorbing layers, effectively concentrating and absorbing solar radiation, rapidly raising the seawater temperature to its evaporation point.^[^ ^13^ ^]^ The resulting steam is then captured and condensed into freshwater. This process, driven entirely by solar energy, substantially reduces the energy consumption typically associated with traditional desalination methods, such as reverse osmosis and multi‐stage flash distillation. Furthermore, the integration of modified lignin materials can enhance evaporation efficiency by providing optimized vapor transport channels, thereby further improving the overall performance and sustainability of the desalination system.

**Figure 7:**
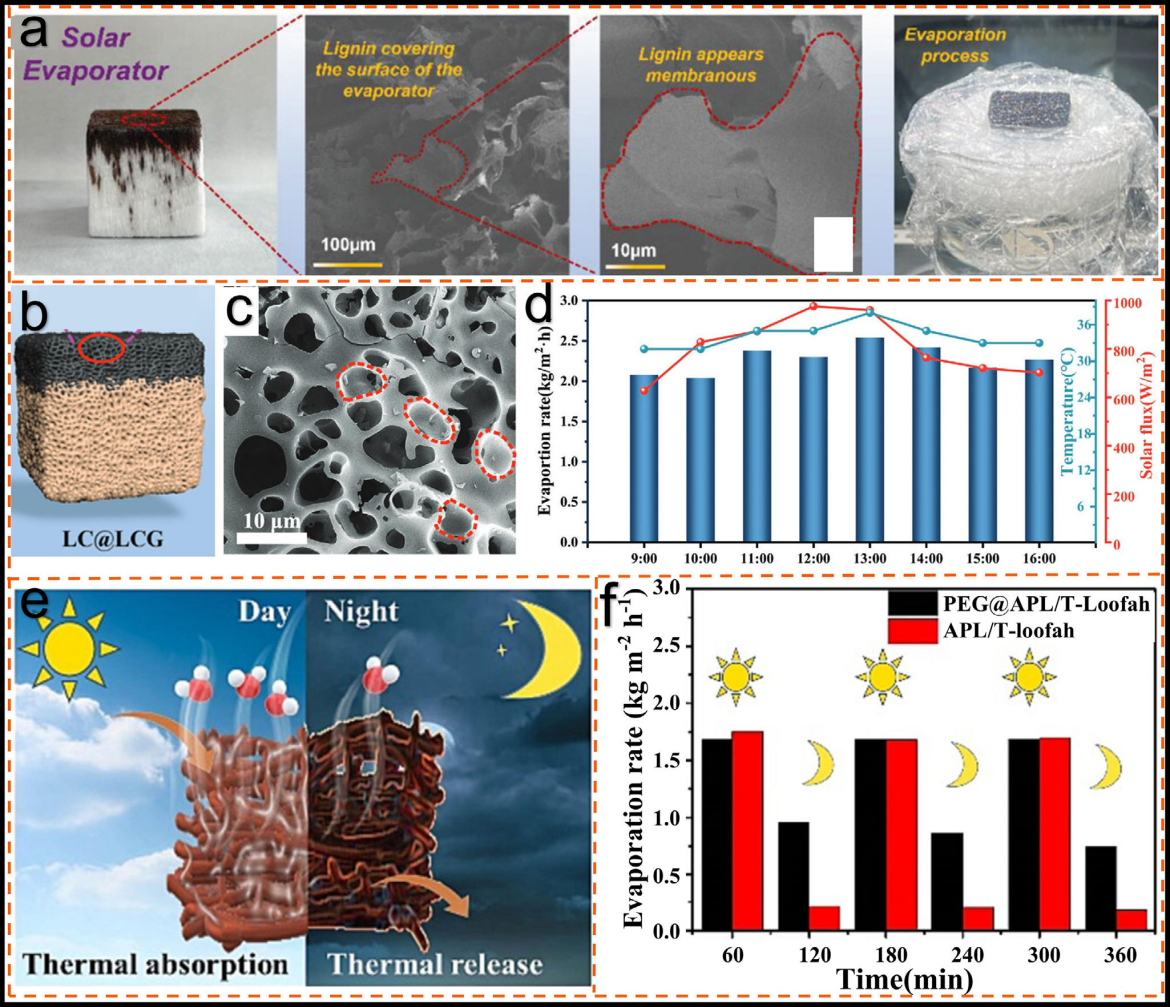
Lignin‐based solar evaporator. a) Image of the sample with the evaporation process and SEM image of the sample.^[^ ^16^ ^]^ Copyright 2023, Wiley. b) Schematic diagram of lignocellulose‐based double‐layered porous hydrogel LC @ LCG.^[^ ^105^ ^]^ Copyright 2022, Wiley. c) SEM images of the porous structure of porous hydrogel LC @ LCG.^[^ ^105^ ^]^ Copyright 2022, Wiley. d) In the outdoor experiments, the rate of water evaporation from the samples with solar intensity and ambient temperature was 9:00 to 17:00.^[^ ^105^ ^]^ Copyright 2022, Wiley. e) Schematic diagram of samples with the ability to be used during the day and night.^[^ ^33^ ^]^ Copyright 2023, Elsevier f) Evaporation rate of the sample during a simulated day and night (light: 1 h, dark: 1 h 3, cycles).^[^ ^33^ ^]^ Copyright 2023, Elsevier.

Gu et al.^[^ ^16^ ^]^ developed a wood‐based solar evaporator that utilizes lignin as the photothermal material, achieving high‐performance steam generation. As shown in **Figure** **7**a, the evaporator and its evaporation process are presented, while the scanning electron microscope image confirms that lignin covers its surface in the form of a thin film. The results show that the addition of lignin not only enhances the structural strength of the evaporator, but also allows for the prevention of salt accumulation through careful adjustment of the lignin content. The optimized lignin‐based solar evaporator exhibits exceptional desalination capacity, dye removal performance and high stability. The full‐spectrum solar absorption rate reached ≈83.6%, the photothermal conversion efficiency reached ≈91.74%, and the evaporation efficiency reached ≈1.93 kg m^−2^h^−1^. These values are far superior to most wood vaporizers. This research presents a new approach for fabricating evaporators with remarkable performance using contain lignin materials.

Lin et al.^[^ ^105^ ^]^ introduced a novel dual‐layer porous hydrogel derived entirely from lignocellulosic biomass for efficient solar steam generation (Figure 7b). The porous carbon material was synthesized by pyrolysis of AL and potassium hydroxide at 800 °C, as shown in Figure 7c, resulting in a loose and uniformly distributed porous structure that facilitates mass transfer. The non‐radiative mobility and photothermal conversion of the hydrogel were enhanced due to the lignin‐rich aromatic ring structure. In outdoor environments, the hydrogel demonstrated excellent evaporation performance, with evaporation rates reaching 2.0–2.5 kg m⁻^2^h⁻¹ in simulated seawater (Figure 7d). These studies present a sustainable and cost‐effective solution for solar steam generation, addressing both freshwater scarcity and environmental challenges. Yue et al.^[^ ^33^ ^]^ reported a highly efficient, all‐biomass solar evaporator based on polyethylene glycol (PEG)‐coated lignin‐decorated loofah sponges. This evaporator achieves a rapid evaporation rate of 1.75 kg·m⁻^2^·h⁻¹ and an efficiency of 97.6% under sunlight, offering exceptional cost‐effectiveness. Lignin materials exhibit significant photothermal effects, attributed to the presence of abundant *π–π* stacking structures. The photothermal conversion process can be precisely controlled by adjusting the number of conjugated structures grafted onto lignin. Additionally, the PEG encapsulation in the evaporator allows for the release of stored excess energy, enabling round‐the‐clock solar desalination of seawater (Figure 7e,f). These investigations provide a sustainable, cost‐efficient solution for solar steam generation, directly addressing the critical issues of freshwater shortage and environmental sustainability.

Although lignin‐based photothermal materials exhibit considerable potential in seawater desalination, they still suffer from various technical and application challenges, such as enhancing material stability and durability, as well as optimizing the overall design and efficiency of the system. Future research will focus on optimizing the performance of lignin‐based photothermal materials and developing robust strategies for their integration into desalination systems, thereby enabling large‐scale deployment. Such efforts are expected to play a pivotal role in unlocking the practical potential of these materials. Looking forward, lignin‐based photothermal materials hold considerable promise for addressing the global issue of freshwater scarcity, while also offering a sustainable energy solution for other industrial sectors.

### 4.4 Biomedical Field

#### 4.4.1 Photothermal Therapy (PTT)

Photothermal therapy (PTT) is a minimally invasive therapeutic strategy that converts light energy into localized thermal energy through photothermal conversion materials, which enables effective treatment of tumors or other diseases.^[^ ^77^, ^106^ ^]^ The excellent biocompatibility and low toxicity of lignin with inherent photothermal conversion capability provide a sustainable promise in advancing photothermal therapy.

**Figure 8:**
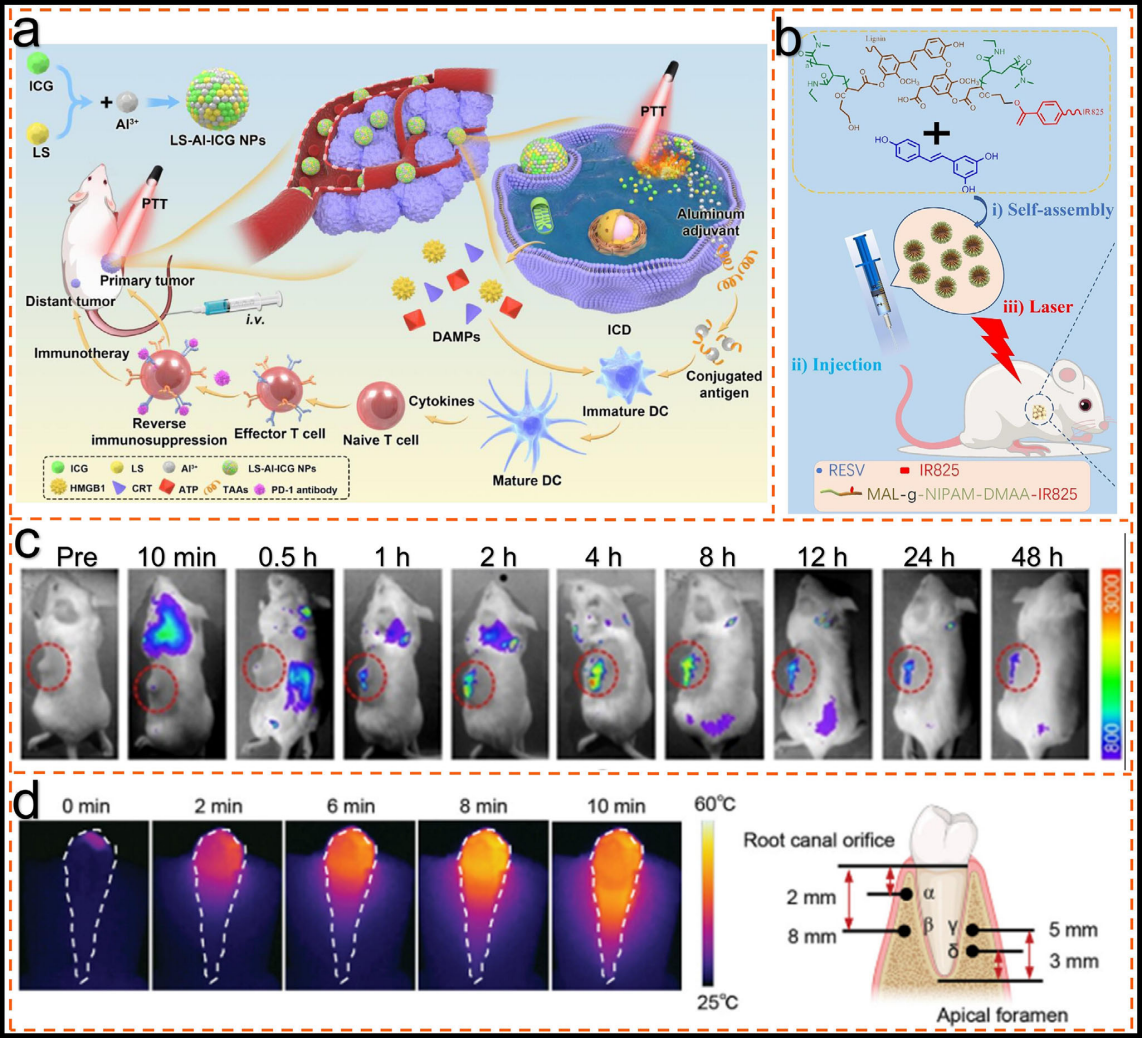
Lignin for photothermal therapy. a). Schematic diagram of lignin composite materials for photothermal immunotherapy of primary and distant breast tumors.^[^ ^17^ ^]^ Copyright 2022, Shenyang Pharmaceutical University. b). Schematic illustration of mnd‐ir@resv micelles for ternary regulation and synergistic therapy: self‐assembly, injection, and tumor elimination upon laser irradiation in mice.^[^ ^26^ ^]^ Copyright 2023, Elsevier c). In vivo realtime photoluminescence imaging after intravenous injection of BPQDs@N‐LgC NPs.^[^ ^107^ ^]^ Copyright 2022, Elsevier d). SLS‐Pd mediated synergistic system for eliminating E. faecalis biofilm in root canals: heat map analysis and schematic diagram of temperature test points.^[^ ^18^ ^]^ Copyright 2022, Wiley.

The excellent biocompatibility and low toxicity of lignin, along with its inherent photothermal conversion capability, offer promising sustainability for advancing photothermal therapy. Lignin can serve as an effective photothermal therapeutic agent due to its natural photothermal conversion properties. When accumulated in tumor tissues, lignin nanoparticles can be activated by near‐infrared (NIR) irradiation, rapidly converting light energy into thermal energy. This localized heating effect raises the temperature within the tumor microenvironment to cytotoxic levels, selectively targeting and eradicating cancer cells while sparing adjacent healthy tissue.^[^ ^5^ ^]^ In addition, the damage to surrounding normal tissues can also be minimized due to its high selectivity. Fan et al.^[^ ^17^ ^]^ utilized a lignin‐assisted approach to construct sub‐10 nm supramolecular assemblies designed for photothermal immunotherapy and enhanced anti‐PD‐1 therapy, effectively targeting both primary and metastatic breast tumors (**Figure** **8**a). Lignin sulfonate (LS), a water‐soluble lignin derivative, was strategically employed as surfactant and stabilizer, enabling its assembly with the photosensitizer indocyanine green (ICG) and the adjuvant aluminum to form the LS‐Al‐ICG nanosystem. This system exhibited enhanced stability through multiple intermolecular interactions, facilitating its selective accumulation within tumor tissues and subsequent degradation within the acidic tumor microenvironment. Under NIR laser irradiation, the LS‐Al‐ICG nanosystem generates a potent photothermal effect, promoting immunogenic cell death and effectively stimulating an antitumor immune response. Furthermore, LS‐based nanocomposites can be synthesized into various morphologies by modulating the pH of the medium, enhancing their adaptability and expanding their potential in targeted drug delivery and cancer therapies.

Tumor microenvironment response and spatiotemporal targeting are emerging strategies in combination therapies, offering the potential for enhanced antitumor efficacy while minimizing side effects. By modulating the TME and achieving precise targeting, therapeutic outcomes can be significantly improved. Peng et al.^[^ ^26^ ^]^ developed a novel, trackable, and specific targeting nanoplatform for CT/PTT/PDT combination therapy, integrating both endogenous and exogenous stimuli (Figure 8b). The system consists of IR825 combined with N‐isopropylacrylamide‐modified lignin (MND‐IR), which encapsulates resveratrol (RESV) (MND‐IR@RESV). MND‐IR@RESV passively accumulates at the tumor site and selectively targets the mitochondria, triggering an endogenous reactive oxygen species (ROS) burst. Lignin modification enhances the nanoplatform's tumor‐targeting ability and synergizes with endogenous stimuli. Tumor‐specific accumulation can be monitored through fluorescence imaging. Furthermore, under the influence of endogenous stimuli, MND‐IR@RESV induces a ROS cycle in the tumor microenvironment, promoting further drug release and triggering apoptosis, thereby producing a synergistic antitumor effect. Upon NIR laser stimulation, MND‐IR@RESV exhibits a strong photothermal effect, raising tissue temperature. This moderate heating enhances cellular uptake, promotes selective accumulation in the tumor, and increases cancer cell death rates. Side effects on normal tissues are minimal, demonstrating the promising potential of this strategy in cancer therapy, particularly highlighting the crucial role of lignin in tumor targeting and synergistic treatment. Liu et al.^[^ ^107^ ^]^ utilized lignin as a sustainable nano‐carrier to construct therapeutic nano‐materials for photodynamic and photothermal therapy. Specifically, the research team grafted the photosensitizer chlorin e6 (Ce6) and mitochondria‐targeted agent triphenylphosphine to the lignin nano‐platform, forming a therapeutic nano‐material named N‐LgC. Ce6 generated singlet oxygen (^1^O_2_) and degraded the lignin's β‐O‐4 aryl ether bonds under light irradiation. The average diameter of the N‐LgC nanoparticles was ≈120 nm and can be degraded into smaller fragments (5–10 nm). N‐LgC exhibited excellent mitochondria‐targeted bioimaging capability and proved effective for photodynamic therapy. Furthermore, N‐LgC was also used as a nano‐carrier for black phosphorus quantum dots (BPQDs) to provide synergistic fluorescence photoacoustic image‐guided photodynamic photothermal therapy for cancer treatment (Figure 8c). Chen et al.^[^ ^18^ ^]^ introduced a metal‐phenol network constructed using polyphenolic substances as stabilizers and reducing agents. Among them, sulfonated lignin‐palladium (SLS‐Pd) exhibits excellent oxidase‐like activity and stable photothermal effect due to ultrafine‐sized palladium nanoparticles and broad NIR absorption properties. The role of sulfonated lignin (SLS) in photothermal effects was mainly reflected in the constructed metal‐phenol network. SLS‐Pd networks are capable of generating heat under NIR light irradiation due to broad NIR absorption and stable photothermal conversion properties. This thermal response significantly elevates the temperature within the irradiated area, producing a reliable photothermal effect. The elevated temperature is believed to enhance the oxidative enzyme‐mimetic activity of the SLS‐Pd network, enabling potent antimicrobial effects, particularly effective against biofilm‐associated infections in the oral cavity (Figure 8d).

In summary, lignin serves as a sustainable nano‐carrier for the construction of therapeutic nano‐materials with photodynamic and photothermal therapy capabilities, paving the way for innovative approaches in cancer treatment and expanding the scope of lignin's applications in biomedical therapeutics.

#### 4.4.2 Photothermal Sterilization

**Figure 9:**
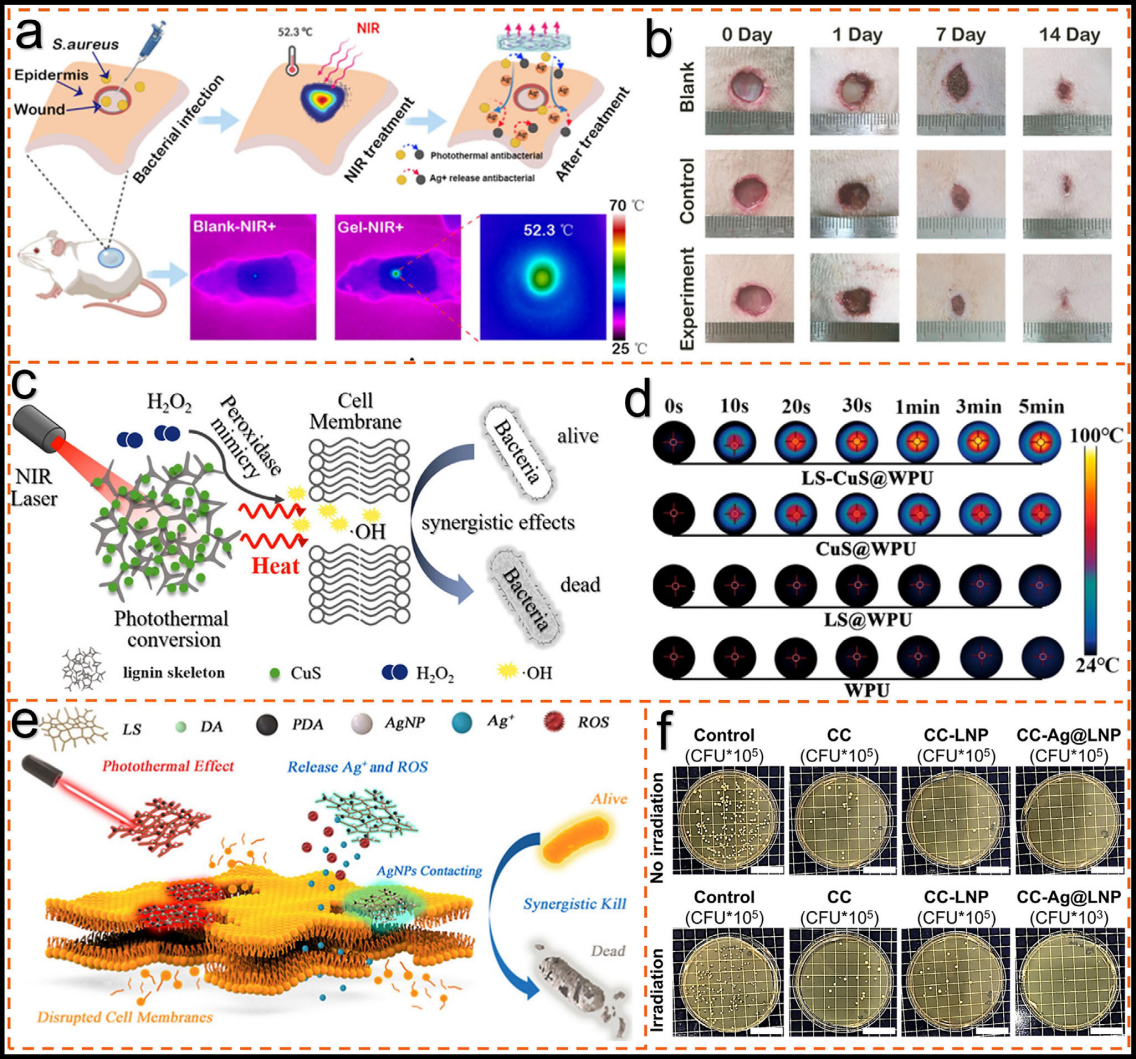
Application of lignin in photothermal sterilization. a) Schematic images of treating infected skin wound by combining Ag‐SLS/PPy‐PDA@PEGDA hydrogel and NIR.^[^ ^109^ ^]^ Copyright 2022, Elsevier. b) Digital photographs of S. aureus infected skin wound 0 day, 1 day, 7 day, and 14 day.^[^ ^109^ ^]^ Copyright 2022, Elsevier. c) Schematic illustrations of the synergistic photothermal catalytic antibacterial mechanism for LS‐CuS nanocomposites.^[^ ^110^ ^]^ Copyright 2021, American Chemical Society. d) Real‐time infrared thermal images of different films under continuous NIR light irradiation (1.8 W cm^−2^) for 5 min. Antimicrobial activity of different composite films against.^[^ ^110^ ^]^ Copyright 2021, American Chemical Society. e) Schematic illustrations of the synergistic bactericidal mechanism of lignin composite materials.^[^ ^31^ ^]^ Copyright 2022, American Chemical Society. f) Digital images of bacterial colonies of *E. coli* after different treatments; scale bar: 3 cm.^[^ ^108^ ^]^ Copyright 2023, Elsevier.

Lignin‐based photothermal materials also show great potential in addressing antibacterial resistance.^[^ ^108^ ^]^ With the escalating issue of antibiotic resistance, the development of innovative antibacterial strategies is urgently needed. L‐NPs absorb light energy at specific wavelengths and convert it into heat, effectively disrupting the microbial cell structure and leading to cell death.^[^ ^19^ ^]^ This approach offers high biocompatibility, selectively targeting microorganisms while minimizing harm to human cells. The photothermal effect allows for precise control over heat generation by adjusting irradiation time and light intensity, thus enabling efficient pathogen eradication through photothermal sterilization. Chen et al.^[^ ^109^ ^]^ designed a synergistic photothermal and antibacterial hydrogel by introducing silver‐sodium lignosulfonate nanoparticles (Ag‐SLS NPs) and polypyrrole‐dopamine nanoparticles (PPy‐PDA NPs) as synergistic photothermal agents into polyethylene glycol diacrylate (PEGDA), preparing Ag‐SLS/PPy‐PDA@PEGDA hydrogel. Benefiting from the ability of Ag and PPy‐PDA NPs to convert NIR light into heat, the resulting hydrogel exhibits ultra‐high photothermal activity and excellent antibacterial properties, effectively combating both Gram‐negative (*Escherichia coli*) and Gram‐positive (Staphylococcus aureus) bacteria. Moreover, the wound healing effect of the Ag‐SLS/PPy‐PDA@PEGDA hydrogel was evaluated using a rat full‐thickness Staphylococcus aureus infection wound model (**Figure** **9**a). After 7 days of treatment, the experimental group showed superior wound healing compared to the other groups, and the hydrogel promoted epidermal regeneration (Figure 9b). This may be due to the effective eradication of most bacteria by NIR treatment, which reduced the inflammatory immune response at the wound site, thereby promoting skin repair and addressing the issue of antibiotic‐free repair of infectious skin defects.

Lignin is considered an ideal carrier for the construction of lignin‐based antimicrobial materials. It has been used as a growth template and stabilizer in the synthesis of lignin‐copper sulfide (LS‐CuS) nanocomposite materials, which exhibit enhanced photothermal properties and peroxidase‐like activity when activated by NIR light. Efficient bactericidal effects has been achieved with nanocomposites through a synergistic photothermal catalytic mechanism (Figure 9c).^[^ ^110^ ^]^ When incorporated into polyurethane films, LS‐CuS nanocomposites form aqueous polyurethane‐based (WPU) composite films that display a pronounced photothermal effect under NIR light exposure. The temperature of the LS‐CuS@WPU film increases rapidly and can maintain a high temperature for up to 5 min, thereby exhibiting stronger antibacterial properties (Figure 9d). This study offers a novel strategy for the environmentally friendly production of lignin‐based nanocomposite materials for use in antimicrobial coatings. Zhang et al.^[^ ^31^ ^]^ introduced photothermal synergistic antibacterial therapy as a new strategy to address antibiotic‐resistant bacterial infections, where lignin was used as a carrier and combined with polydopamine and silver nanoparticles to form a composite material (Figure 9e). These composite materials generate photothermal effect under NIR, effectively converting absorbed light energy into heat energy, which significantly enhances the antimicrobial properties of the composite for efficient sterilization. Liu et al.^[^ ^108^ ^]^ proposed an antibacterial film based on sustainable resources and production methods. This biobased nanocomposite film is composed of chitosan, LNPs, and trace silver nanoparticles (AgNPs). The nanocomposite film is capable of absorbing 89% of the full solar spectrum radiation and exhibits significant photothermal‐triggered antibacterial effects, with the dark color of lignin further enhancing this effect. Under simulated sunlight exposure, the nanocomposite film significantly reduced the survival of *Escherichia coli* compared to the control group (Figure 9f). This nanocomposite material has broad application potential in sunlight‐activated antibacterial films and coatings, meeting the growing demand for sustainable and effective antibacterial materials.

The potential of lignin‐based photothermal materials in biomedical applications, particularly in photothermal therapy and sterilization, provides compelling evidence for their utility in the field. Due to their natural abundance, cost‐effectiveness, biocompatibility, and low toxicity, lignin‐based materials are highly attractive candidates for medical and healthcare applications. Nevertheless, further improvements in the stability, targeting efficiency, and photothermal conversion efficiency of lignin remain essential challenges for current research. As advancements in nanotechnology and bioengineering continue to evolve, lignin and its derivatives are poised to play an increasingly impactful role in medical and healthcare innovations, facilitating the development of sustainable and effective therapeutic and antibacterial solutions.

### 4.5 Other Applications

Lignin photothermal materials exhibit broad applications in pollution treatment, photothermal phase change materials, thermoelectric conversion, photothermal deicing, and thermal management.^[^ ^12^, ^79^, ^100^, ^111^ ^]^ The underlying mechanism is primarily based on lignin's inherent ability to convert light energy into heat, which drives the functionality of these materials. In pollution treatment, lignin's photothermal properties enable the degradation of pollutants upon light irradiation, facilitating an environmentally friendly cleanup process. For photothermal phase change materials, the photothermal conversion of lignin contributes to efficient thermal energy storage by enabling phase transitions at desired temperatures. In thermoelectric conversion, the thermal energy generated by lignin can enhance energy harvesting efficiency. In the case of photothermal deicing, lignin's light‐to‐heat conversion facilitates the melting of ice and snow, improving safety and operational efficiency in cold environments. Lastly, in thermal management, lignin is utilized to regulate temperature by controlling heat distribution within systems. These applications fully demonstrate the unique advantages of lignin in harnessing photothermal energy across various fields.

**Figure 10:**
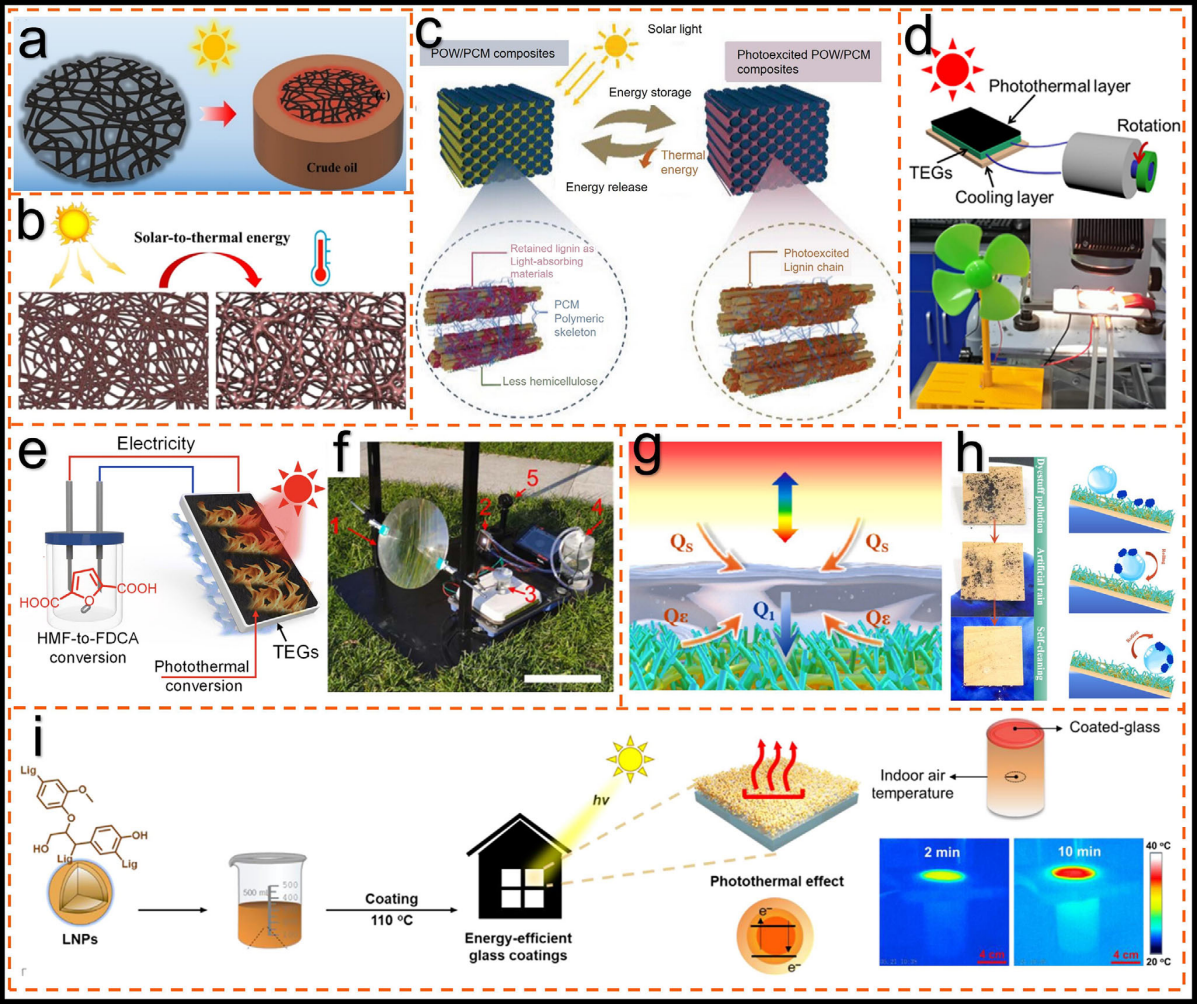
The application of lignin‐based photothermal materials in pollution treatment, phase change materials, photothermal power generation, photothermal power generation coupled with electrocatalysis, photothermal deicing, and thermal management. a) Schematic diagram of crude oil absorption by lignin composite membrane.^[^ ^30^ ^]^ Copyright 2022, Elsevier. b) Solar‐to‐thermal energy conversion and storage procedure under sunlight.^[^ ^112^ ^]^ Copyright 2022, American Chemical Society. c) Photothermal energy storage and conversion.^[^ ^113^ ^]^ Copyright 2023, Wiley. d) Schematic illustration for the solar thermal generator and the voltage of TEGS driven by solar energy (xenon lamp).^[^ ^12^ ^]^ Copyright 2022, American Chemical Society. e) The solar–thermal–electrocatalytic process for the integrated biorefinery.^[^ ^5^ ^]^ Creative Commons Attribution 3.0 Unported Licence. f) Images of the setup for an authentic field experiment. 1) Fresnel lens; 2) TEGs; 3) quartz reactor; 4) cooling system; 5) solar meter. Scale bar = 20 cm.^[^ ^5^ ^]^ Creative Commons Attribution 3.0 Unported Licence. g) Self‐cleaning tests of F–Al–Si‐LNP‐4.^[^ ^34^ ^]^ Copyright 2024, Elsevier. h) Schematic diagram of self‐cleaning.^[^ ^34^ ^]^ Copyright 2024, Elsevier. i) Fully biobased photothermal films and coatings for indoor ultraviolet radiation.^[^ ^117^ ^]^ Copyright 2022, The Authors. This publication is licensed under CC‐BY 4.0.

Lignin‐based photothermal materials have also garnered significant attention for their application in environmental pollution control, especially in responding to emergency environmental pollution events, phase change materials, photothermal power generation, photothermal deicing, and thermal management. The photothermal conversion properties of lignin allow it to quickly heat up under sunlight, promoting the adsorption and removal of oil pollutants on its surface. Wei et al.^[^ ^30^ ^]^ developed polylactic acid‐modified lignin (PLA) particles combined with dopamine into the fabrication of photothermal films. The synergistic photothermal effect of PLA‐modified lignin as a support scaffold and dopamine makes the photothermal films exhibit excellent photothermal conversion ability. The resulting film could effectively reduce the viscosity of crude oil under sunlight and is used to repair crude oil spills (**Figure** **10**a). Solar energy can be maximized under the synergistic effect of lignin composites’ highly efficient photothermal effect to reduce electricity consumption and fossil fuel use, which contributes to alleviating freshwater shortages, removing oil pollution and further achieving carbon neutrality.

Organic phase change materials (PCMs) hold great promise for converting thermal energy from solar radiation into photothermal energy. However, issues such as poor light absorption and liquid leakage have significantly limited their practical application. Niu et al.^[^ ^112^ ^]^ developed lignin‐based phase change nanofiber films (PCNFs) tailored for solar energy storage. In this design, lignin serves as a photothermal agent, facilitating non‐radiative energy transfer via *π–π* stacking interactions among lignin molecules, thereby enabling efficient photothermal conversion (Figure 10b). Furthermore, lignin's structural integrity reinforces the PCNFs through robust hydrogen bonding and molecular entanglement with polymers, effectively preventing leakage and transfer issues associated with phase‐change materials (PCMs). Additionally, lignin's function as an anionic surfactant promotes polymer bonding during electrospinning, further enhancing solar energy conversion and storage capabilities. In related work, Yang et al.^[^ ^113^ ^]^ demonstrates a sustainable porous scaffold in which high‐iodine wood (POW) serves as the supporting material, while lignin is in situ retained as a light‐absorbing dopant (Figure 10c). The *π–π* stacking interactions of lignin molecules enable efficient light energy absorption, promoting rapid heat conductivity and resulting in a higher maximum energy storage capacity. The natural porous structure of the POW scaffold ensures excellent shape stability, effectively addressing the issue of liquid leakage. Additionally, the retention of lignin enhances material stability and durability, allowing the composite to maintain good performance after multiple thermal cycles. Lignin enhances the photothermal conversion efficiency of the composite material and strengthens its potential as a thermal energy storage material, thereby offering increased possibilities for future practical applications.

Lignin‐based photothermal materials have garnered significant attention in the field of energy conversion due to their exceptional photothermal conversion performance. In particular, lignin shows considerable promise in photothermal power generation applications.^[^ ^12^ ^]^ In photothermal power generation systems, the photothermal capabilities of lignin‐based materials can be converted into electricity via a thermoelectric generator (TEG). In this system, lignin photothermal materials absorb sunlight, converting it into thermal energy, which is then transformed into electrical energy by the TEG. The TEG operates based on the principle of generating electricity from a temperature difference.^[^ ^114^ ^]^ When the lignin photothermal material is exposed to sunlight, its surface temperature rises, creating a temperature gradient that drives the TEG, thereby converting light energy into electrical energy. Zhao et al.^[^ ^12^ ^]^ investigated lignin‐derived LNPs as a low‐cost, efficient solar thermal material for converting solar energy into electricity. The photothermal conversion efficiency of L‐NPs is 22%, and their photothermal conversion capability is attributed to the inherent *π−π* stacking interactions of lignin molecules. Under artificial solar radiation, L‐NPs successfully drove a TEG (Figure 10d). This study presents a novel strategy for the use of lignin in photothermal materials, enabling the conversion of light energy into electrical energy. Liu et al.^[^ ^115^ ^]^ developed a biphasic solvent system composed of lithium chloride tetrahydrate and γ‐valerolactone (GVL) to selectively separate hemicellulose and lignin from lignocellulose. The isolated lignin was then fully converted into a photothermal material and coordinated with iron ions for solar thermoelectric conversion. This innovative approach facilitates the direct utilization of lignin‐derived products in the fabrication of functional materials, thereby overcoming limitations commonly encountered in conventional lignin biorefinery processes. Further utilizing the photothermal conversion capability of lignin to provide electricity for biomass refining, Zhao et al.^[^ ^5^ ^]^ explored a novel solar photothermal‐electrocatalytic process in lignin‐based biomass refining, achieving the efficient conversion of 5‐hydroxymethylfurfural (HMF) to 2,5‐furandicarboxylic acid (FDCA) (Figure 10e). After the methyl groups of lignin were removed, they coordinated with Fe^3+^ to form D‐Lig‐Fe, exhibiting high photothermal conversion efficiency. Under natural sunlight and assisted by a Fresnel lens (Figure 10f), the photothermal conversion process drove a TEG to generate electricity, which, in turn, enabled the selective and efficient conversion of HMF to FDCA under the action of a NiCoB catalyst. This provides a new technological pathway for energy generation and high‐value chemical production in lignin‐based biomass refining. As research advances, lignin‐based photothermal materials demonstrate expanding potential in photothermal power generation. These materials contribute significantly to both sustainable energy development and environmental technology, underscoring their considerable promise.

In colder climates, ice and snow accumulation on equipment surfaces can severely impair efficiency or cause damage.^[^ ^116^ ^]^ Utilizing lignin's photothermal conversion capability, light‐induced heating can generate localized warmth on ice‐covered surfaces, rapidly melting the ice layer and enabling de‐icing. Wu et al.^[^ ^34^ ^]^ prepared from a deep eutectic solvent of sulfonamide acid and urea, creating a multi‐level roughness structure with LNPs, bauxite, and silica sol to form a UV‐shielding protective coating on the surface of poplar veneer. The phenolic hydroxyl groups, carbonyl groups, and conjugated double bonds in LNPs enable excellent UV shielding properties through *π–π* stacking interactions, allowing the modified poplar veneer to strongly absorb ultraviolet A (UVA) radiation. The side chains of LNPs, when combined with amines, enhance the internal conjugation effect, promoting the photothermal effect (Figure 10g). The layered structure of the composite material extends the light propagation path, improving the photothermal conversion efficiency. The heat generated by the photothermal conversion of LNPs enables the modified poplar veneer to exhibit photothermal de‐icing properties. Moreover, the superhydrophobicity and durability of the composite allow the poplar veneer to achieve self‐cleaning through hydration (Figure 10h), extending its service life in harsh outdoor environments. With ongoing technological advancements, the efficiency and stability of lignin‐based photothermal de‐icing materials are expected to improve further, driving widespread application in fields such as aerospace, transportation, and energy equipment.

Lignin‐based photothermal materials also show significant potential in indoor temperature management. Liu et al.^[^ ^117^ ^]^ reported fully biobased nanocomposite films and coatings with high photothermal activity and selective UV radiation absorption. These nanocomposites contain 20 wt% LNPs embedded in a chitosan matrix, effectively blocking 97% of UV radiation at a wavelength of 400 nm while exhibiting excellent solar energy collection properties. The reflection spectrum of the nanocomposite films indicates that the uniform dispersion of nanoparticles within the matrix is crucial for efficient UV blocking. Furthermore, the study demonstrated that nanocomposites containing 20 wt% LNPs could be used as photothermal glass coatings for passive cooling of indoor environments (Figure 10i). By adjusting the coating thickness, a 20 µm layer can reduce temperature rise by 58% under simulated solar radiation, showing a significant cooling effect compared to uncoated glass systems. These renewable nanocomposite films and coatings offer a sustainable approach to indoor thermal management, enhancing human health and well‐being while highlighting lignin's potential as an eco‐friendly material for energy‐saving and temperature regulation applications.

Collectively, these studies highlight the diverse applications of lignin‐based photothermal materials, suggesting their versatility and substantial promise in advancing sustainable, high‐performance materials for future technologies.

## 5 Conclusion 

This review explores lignin‐based photothermal materials as a renewable resource, highlighting their considerable potential in tackling the global energy and environmental crises. These materials offer promising advancements in applications such as seawater desalination, photothermal therapy, photocatalysis, and energy storage, while simultaneously providing sustainable solutions to the escalating environmental challenges. Their widespread use is poised to play a pivotal role in addressing the urgent issues of global energy shortages and environmental pollution. This review provides an in‐depth examination of lignin‐based photothermal materials, detailing fundamental concepts, classifications, design strategies, and various applications. Noteworthy for its abundance, cost‐effectiveness, and intrinsic photothermal properties, lignin holds significant promise for applications in desalination, photothermal therapy, actuation, and energy storage. Research into lignin‐based photothermal materials reveals both promising opportunities and complex challenges. Developing green and efficient strategies to optimize these materials is essential for expanding their applicability across energy, environmental, and biomedical domains. Emerging areas such as photothermal catalysis and responsive smart materials are also driving new research directions, highlighting the versatility and adaptability of lignin‐based solutions. To fully leverage the potential of lignin‐based photothermal materials, advancing scalable production techniques and fostering commercialization will be critical. In conclusion, lignin photothermal materials stand out for their unique blend of sustainability and functional versatility, marking them as vital resources in the pursuit of sustainable energy solutions and environmental management. Their advancement represents an important step toward addressing global challenges through innovative materials science.

## 6 Challenges and Future Outlook

Lignin, as a renewable biomass resource, holds immense potential as a photothermal material. Despite significant progress at the laboratory scale, its commercialization and large‐scale production still face several key challenges. In order to promote the practical application of lignin‐based photothermal materials, in‐depth research, and optimization in several areas are required, particularly regarding production costs, technical bottlenecks, and issues encountered in real‐world applications.

1) Challenges in production costs and optimization of extraction methods: Although lignin can be extracted from wood, agricultural waste, and industrial by‐products, the current extraction processes, particularly the kraft process, although capable of achieving high yields, are accompanied by significant environmental pollution, complex procedures, and high costs.

Although the extraction cost of lignin is high, its main sources are industrial by‐products such as pulp and paper waste, agricultural residues (e.g., straw, rice husks), and biomass energy by‐products, providing an opportunity for waste utilization. This recycling approach reduces extraction costs and minimizes resource waste, supporting the circular economy. Future research should focus on developing low‐cost, low‐pollution extraction methods, such as enzymatic hydrolysis or green solvents, to improve lignin recovery efficiency and reduce environmental impact. Optimizing solvent recovery to minimize resource waste is also crucial for lowering production costs.

2) Technological bottlenecks and standardization pathways for large‐scale production: Lignin‐based photothermal materials have demonstrated excellent performance at the laboratory scale, consistency and stability issues persist in large‐scale production. The variety of plant sources for lignin, along with the significant differences in molecular structure, molecular weight, and functional group distribution across different plants, complicates raw material processing and consumes considerable time during large‐scale production.

To improve the efficiency and consistency of large‐scale lignin production, technological measures must optimize its molecular structure and extraction processes. Optimizing fractionation and separation technologies is crucial for addressing consistency and stability issues. Fractionation can precisely separate and purify lignin based on properties like molecular weight and solubility, ensuring uniform quality and better control of physical properties across batches. Standardized pretreatment methods, such as high‐temperature hydrolysis or acid‐base catalysis, help overcome structural differences in lignin from various plant sources, reducing variability and ensuring consistent molecular structure. These methods also remove impurities and break down molecular aggregates, enhancing lignin's performance in photothermal materials.

3) Functional modification and feasibility for long‐term applications: Lignin‐based photothermal materials have shown excellent laboratory performance but face challenges in long‐term stability and durability in practical applications. Lignin's limited active sites and low chemical reactivity hinder its compatibility with other materials, restricting its long‐term use. Additionally, under high‐temperature or complex conditions, the molecular structure may degrade, reducing photothermal conversion efficiency.

To enhance lignin's long‐term performance, functional modifications are necessary. Increasing its crosslinking density strengthens its chemical activity and compatibility, improving structural stability. Since lignin's amphiphilic nature often causes phase separation, developing a cosolvent system can improve solubility and dispersion, enhancing compatibility with other materials. Using green solvents like acetic acid or glycerol, or a combination of polar and non‐polar solvents, helps form a uniform dispersion, improving both photothermal efficiency and material durability. conclusion, the commercialization of lignin‐based photothermal materials faces key challenges such as high production costs, environmental concerns, and market demand. Overcoming these requires optimizing the industrial value chain, integrating lignin production with photothermal material manufacturing, and ensuring compliance with sustainable practices.

In conclusion, the commercialization of lignin‐based photothermal materials faces key challenges such as high production costs, environmental concerns, and market demand. Overcoming these requires optimizing the industrial value chain, integrating lignin production with photothermal material manufacturing, and ensuring compliance with sustainable practices.

## Conflict of Interest

The authors declare no conflict of interest.
